# Spectral Flow Cytometry Methods and Pipelines for Comprehensive Immunoprofiling of Human Peripheral Blood and Bone Marrow

**DOI:** 10.1158/2767-9764.CRC-23-0357

**Published:** 2024-03-25

**Authors:** Milos Spasic, Esther R. Ogayo, Adrienne M. Parsons, Elizabeth A. Mittendorf, Peter van Galen, Sandra S. McAllister

**Affiliations:** 1 Division of Hematology, Department of Medicine, Brigham and Women's Hospital, Boston, Massachusetts.; 2 Department of Medicine, Harvard Medical School, Boston, Massachusetts.; 3 Division of Breast Surgery, Department of Surgery, Brigham and Women's Hospital, Boston, Massachusetts.; 4 Breast Oncology Program, Dana-Farber Brigham Cancer Center, Boston, Massachusetts.; 5 Broad Institute of Harvard and MIT, Cambridge, Massachusetts.; 6 Harvard Stem Cell Institute, Cambridge, Massachusetts.; 7 Ludwig Center at Harvard, Harvard Medical School, Boston, Massachusetts.

## Abstract

Profiling hematopoietic and immune cells provides important information about disease risk, disease status, and therapeutic responses. Spectral flow cytometry enables high-dimensional single-cell evaluation of large cohorts in a high-throughput manner. Here, we designed, optimized, and implemented new methods for deep immunophenotyping of human peripheral blood and bone marrow by spectral flow cytometry. Two blood antibody panels capture 48 cell-surface markers to assess more than 58 cell phenotypes, including subsets of T cells, B cells, monocytes, natural killer (NK) cells, and dendritic cells, and their respective markers of exhaustion, activation, and differentiation in less than 2 mL of blood. A bone marrow antibody panel captures 32 markers for 35 cell phenotypes, including stem/progenitor populations, T-cell subsets, dendritic cells, NK cells, and myeloid cells in a single tube. We adapted and developed innovative flow cytometric analysis algorithms, originally developed for single-cell genomics, to improve data integration and visualization. We also highlight technical considerations for users to ensure data fidelity. Our protocol and analysis pipeline accurately identifies rare cell types, discerns differences in cell abundance and phenotype across donors, and shows concordant immune landscape trends in patients with known hematologic malignancy.

**Significance:**

This study introduces optimized methods and analysis algorithms that enhance capabilities in comprehensive immunophenotyping of human blood and bone marrow using spectral flow cytometry. This approach facilitates detection of rare cell types, enables measurement of cell variations across donors, and provides proof-of-concept in identifying known hematologic malignancies. By unlocking complexities of hematopoietic and immune landscapes at the single-cell level, this advancement holds potential for understanding disease states and therapeutic responses.

## Introduction

Assessment of hematopoietic and peripheral blood cell counts and morphology provides a vital source of information in both research and clinical settings. For example, complete blood counts, white blood cell differentials, and blood smears have long played an important role in standard clinical diagnoses (1). More recently, the integration of immunoprofiling into translational research has enabled characterization of cell abundance and phenotypes that reflect disease risk and disease status, identification of prognostic and predictive biomarkers of therapeutic response, and development of new therapies (2–5). For example, an immune signature generated from analysis of peripheral blood mononuclear cell (PBMC) immunophenotypes was shown to correlate with all-cause mortality (2).

Flow cytometry is a widely used immunophenotyping technique that enables analysis of single-cell protein expression using fluorochrome-labeled antibodies that bind to those proteins. Early versions of flow cytometers could analyze up to eight cell-surface markers simultaneously. With advancements in technology, modern flow cytometers can now accommodate a larger number of markers (6–11). Despite those improvements, spectral overlap between fluorophores presents limitations to deeper immunophenotyping (12). The development of mass cytometry, which uses metal-conjugated antibodies, has expanded the number of markers that can be simultaneously assessed (13–15). Mass cytometry enables high parameter analysis by utilizing metal-conjugated antibodies instead of fluorophores (16, 17); however, the adoption of mass cytometry is complicated by low sample throughput and high cost (12).

Full spectrum, or spectral, flow cytometry seeks to address both the spectral limitations of conventional flow cytometry and the cost limitations of mass cytometry (5, 18, 19). Spectral flow cytometry leverages the entire emission spectrum of a given fluorophore, thus capturing its unique spectral signature. Fluorophores are distinguished from one another by their spectral signatures, even when their maximum emissions peaks are similar. This technology enables the use of highly multiparametric antibody-fluorophore cocktails that can be combined in a single sample tube to provide better resolution of cell types in complex tissues.

Here, we present two human PBMC panels and a bone marrow cell (BMC) panel, developed and validated using a four-laser full spectrum flow cytometer. Our PBMC antibody panels identify 48 cell-surface markers to assess >58 peripheral immune cells and their functional states in less than 2 mL of a donor blood sample. Our BMC antibody panel captures 32 cell-surface markers for granular analysis of hematopoietic and immune cells in a single donor sample. Incorporating a high number of markers allows us to detect cell types (e.g., T cells), their subtypes (e.g., effector memory T cells), and differentiation and activation states (e.g., exhaustion). We also implement data visualization and batch correction strategies typically used for single-cell sequencing. Our pipeline successfully identified rare cell types, variances in cell abundance and phenotype among donors, and identified individuals known to be diagnosed with hematologic malignancies. While this resource is not yet intended as a diagnostic tool, it represents a straightforward, efficient, and adaptable pipeline for comprehensive immunoprofiling and biological discovery, setting a new standard for high-resolution and high-throughput immunologic studies.

## Materials and Methods

### Experimental Model and Subject Details

Blood donors were selected from patients being followed for a high-risk breast lesion at the Brigham and Women's Hospital Breast Cancer Personalized Risk Assessment Prevention and Education Program (B-PREP). Written informed consent was obtained from patients before sample collection allowing for use of samples for research. The use of human samples was approved by the Dana-Farber/Harvard Cancer Center Institutional Review Board. All procedures performed in this study involving human participants were in accordance with the ethical standards of the institutional and/or national research committee and with the 1964 Helsinki Declaration and its later amendments. All blood donors were non-Hispanic White females, and investigators were blinded to patient age, weight, and potential comorbidities prior to final data analysis. Bone marrow cells were purchased as cryopreserved vials from Lonza (catalog no. 2M-125C). Donors consented to all study procedures.

### Antibodies and Panel Design

Detailed information for all antibodies in this study is provided in Tables 1 and 2, and Supplementary Tables S1, S2, and S3. Antibody staining panels were designed and validated on a four-laser (405, 488, 561, and 640 nm) Cytek Aurora (Cytek Biosciences) as described previously (18). Briefly, to identify the best possible fluorochrome combinations for all three panels, fluorochrome signature uniqueness was first determined by comparing the full spectrum across all 48 detectors and quantified using the similarity index metric developed by Cytek Biosciences. This index ranges from 0 to 1; 0 indicating that two fluorochromes do not share any spectral characteristics, and 1 indicating identical spectra (18). On the basis of testing of multiple fluorochrome combinations, it was determined that similarity indices of 0.98 or less indicated that fluorochromes were different enough to be used together. Next, overall fluorochrome combination compatibility was assessed using the complexity index, a metric also developed by Cytek Biosciences. This index measures the interference among fluorochrome combinations and predicts the impact on the autofluorescence distribution of the spectrally unmixed results while also considering spillover (18). The lower the complexity index, the higher the probability that the fluorochrome combination will efficiently yield high resolution data through reduced spread. Both similarity and complexity index metrics are available through the SpectroFlo software. On the basis of the above criteria, 27 fluorochromes were selected for the T-cell and B-cell panel (T/B panel; complexity index = 13.11), 25 fluorochromes for the monocytic and myeloid cell, natural killer T (NKT) cell, NK cell, dendritic cell (DC; M/N/D panel; complexity index = 12.82), and 32 fluorochromes for the BMC panel (complexity index = 31.16).

**TABLE 1:** PBMC panels markers. Cell-surface markers used to gate and identify indicated cell types in PBMC samples

Major cell population	Subsets	Gating	Reference
**T/B Panel**			
**Total T cells**		**CD45^+^/CD3^+^**	**PMID: 32830910**
**CD8^+^ T cells**		**CD45^+^/CD3^+^/CD4^−^/CD8^+^**	**PMID: 32830910**
	Naïve CD8	CD45RA^+^/CD197^+^	PMID: 24258910; PMID: 10537110; PMID: 28069807; PMID: 18657274
	TEMRA CD8	CD45RA^+^/CD197**^−^**	PMID: 10537110; PMID: 28069807; PMID: 18657274
	CD45RA terminal effector CD8	CD45RA^+^/CD197**^−^**/CD27^−^/CD28**^−^**	PMID: 18657274
	Central memory CD8	CD45RA^−^/CD197^+^	PMID: 24258910; PMID: 10537110; PMID: 28069807; PMID: 18657274
	Effector CD8	CD45RA**^−^**/CD197**^−^**	PMID: 24258910; PMID: 10537110; PMID: 28069807; PMID: 18657274
	Early effector CD8	CD45RA**^−^**/CD197**^−^**/CD28^+^/CD27^+^	PMID: 18785267; PMID: 18657274
	Early-like effector CD8	CD45RA**^−^**/CD197**^−^**/CD28^+^/CD27^−^	PMID: 18785267; PMID: 18657274
	Terminal effector CD8	CD45RA^−^/CD197^−^/CD28^−^/CD27^−^	PMID: 18785267; PMID: 18657274
	Intermediate effector CD8	CD45RA**^−^**/CD197**^−^**/CD28^−^/CD27^+^	PMID: 18785267; PMID: 18657274
**CD4^+^ T cells**		**CD45^+^/CD3^+^/CD8** ^−^ **/CD4^+^**	**PMID: 32830910**
	Naïve CD4	CD45RA^+^/CD197^+^	PMID: 24258910; PMID: 10537110; PMID: 28069807; PMID: 18657274
	TEMRA CD4	CD45RA^+^/CD197**^−^**	PMID: 10537110; PMID: 28069807; PMID: 18657274; PMID: 29133794
	CD45RA terminal effector CD4	CD45RA^+^/CD197**^−^**/CD27**^−^**/CD28**^−^**	PMID: 18657274; PMID: 29133794
	Central memory CD4	CD45RA**^−^**/CD197^+^	PMID: 24258910; PMID: 10537110; PMID: 28069807; PMID: 18657274
	Follicular helper T	CD45RA**^−^**/CD197^+^/CD185^+^/CD183all	PMID: 30714682
	Effector CD4	CD45RA^−^/CD197^−^	PMID: 24258910; PMID: 10537110; PMID: 28069807; PMID: 18657274
	Early effector CD4	CD45RA**^−^**/CD197**^−^**/CD28^+^/CD27^+^	PMID: 18785267; PMID: 18657274
	Early-like effector CD4	CD45RA**^−^**/CD197**^−^**/CD28^+^/CD27**^−^**	PMID: 18785267; PMID: 18657274
	Terminal effector CD4	CD45RA**^−^**/CD197**^−^**/CD28**^−^**/CD27**^−^**	PMID: 18785267; PMID: 18657274
	Intermediate effector CD4	CD45RA**^−^**/CD197**^−^**/CD28**^−^**/CD27^+^	PMID: 18785267; PMID: 18657274
	Regulatory T cells	CD25^+^/CD127^−^	PMID: 23166007
	Th1	CD183^+^/CD196**^−^**	PMID: 33219622; PMID: 30714682
	Th2	CD194^+^/CD196**^−^**	PMID: 33219622; PMID: 30714682
	Th9/22	CD183^−^/CD196^+^/CD194^+^	PMID: 30714682
	Th17	CD161^+^/CD196^+^	PMID: 30714682
**Gamma delta T cells**		**CD45^+^/CD3^+^/TCRγδ^+^**	**PMID: 28119690;** **PMID: 32830910**
	CD197**^−^**/CD45RA^++^/TCRgd^+^	CD197**^−^**/CD45RA^++^	PMID: 12900516; PMID: 32830910
	CD197^+^/CD45RAall/TCRgd^+^	CD197^+^/CD45RAall	PMID: 12900516; PMID: 32830910
	CD197**^−^**/CD45RA**^−^**/TCRgd^+^	CD197**^−^**/CD45RA**^−^**	PMID: 12900516; PMID: 32830910
**Activated T cells**		**CD45^+^/CD3^+^/HLA**^−^**DR^+^/CD38^+^**	**PMID: 26362266; PMID: 29483513**
**NKT-like T cells**		**CD45^+^/CD3^+^/TCRγδ**^−^**/CD161^+^**	**PMID: 21787777**
**NK cells**		**CD45^+^/CD3**^−^**/TCRγδ**^−^**/TCRαβ^−^/HLA-DR** **^−^**	**PMID: 32830910**
**Total B cells**		**CD45^+^/CD19^+^**	**PMID: 28715846**
	Plasmablasts	CD27^+^/CD38^+^	PMID: 28715846; PMID: 15778361
	CD20^+^ B cells	CD20^+^	PMID: 28715846
	Naïve	CD20^+^/CD27**^−^**/IgD^+^	PMID: 28715846; PMID: 9802980
	Switched memory B	CD20^+^/CD27^+^/IgD**^−^**	PMID: 28715846; PMID: 9802980
	Unswitched memory B	CD20^+^/CD27^+^/IgD^+^	PMID: 28715846; PMID: 9802980
	DN memory B	CD20^+^/CD27**^−^**/IgD**^−^**	PMID: 28715846; PMID: 9802980
**Checkpoint inhibitors**	**PD-1**	**CD279^+^**	**PMID: 32429929**
	TIM-3	CD366^+^	PMID: 32429929
	LAG-3	CD223^+^	PMID: 32429929
	CTLA-4	CD152^+^	PMID: 32429929
**M/N/D Panel**			
**NKT cells**		**CD45^+^/CD3^+^/CD56^+^**	**PMID: 24779018**
**NK cells**		**CD45^+^/CD3^−^/CD19^−^/CD14^−^/CD7^+^**	**PMID: 19805616**
	Early NK cells	CD56^+^/CD16**^−^**	PMID: 23650273; PMID: 32830910
	Mature NK cells	CD56^+^/CD16^+^	PMID: 23650273; PMID: 32830910
	Terminal NK cells	CD56**^−^**/CD16^+^	PMID: 23650273; PMID: 32830910
**NK cell status**	**KLRD1**	**CD94^+^**	**PMID: 23650273**
	NKG2D	CD314^+^	PMID: 23650273
	NKG2C	CD159c^+^	PMID: 23650273
	NKp30	CD337^+^	PMID: 23650273
	NKp46	CD335^+^	PMID: 23650273
	NKp44	CD336^+^	PMID: 23650273
	KIR2DL1/S1/S3/S5	CD158^+^	PMID: 23650273
	TIM-3	CD366^+^	PMID: 32858904
**Classical monocytes**		**CD45^+^/CD14^+^/CD16^−^**	**PMID: 20628149**
	Classical monocytes-Normal	HLA-DR^+^	PMID: 31191529
	Classical monocytes-Immunosuppressed	HLA-DR**^−^**	PMID: 31191529
**Intermediate monocytes**		**CD45^+^/CD14^+^/CD16^+^**	**PMID: 20628149**
	Intermediate monocytes-Normal	HLA-DR^+^	PMID: 31191529
	Intermediate monocytes-Immunosuppressed	HLA-DR**^−^**	PMID: 31191529
**Nonclassical monocytes**		**CD45^+^/CD14^−^** **/CD16^+^**	**PMID: 20628149**
	Nonclassical monocytes-Normal	HLA-DR^+^	PMID: 31191529
	Nonclassical monocytes-Immunosuppressed	HLA-DR**^−^**	PMID: 31191529
**MDSC-like cells**		**CD45^+^/CD11b^+^/CD33^+^/HLA-DR^−^**	**PMID: 28936253**
	Monocytic MDSC-like cells	CD14^+^	PMID: 28936253
**Dendritic cells**		**CD45^+^/CD3^−^/HLA-DR^+^/CD19^−^/CD20^−^**	**PMID: 29356334**
	cDC1	CD14**^−^**/CD123**^−^**/CD141^+^/CD1c**^−^**/CD5**^−^**	PMID: 29356334, PMID: 31474513
	cDC2	CD14**^−^**/CD123**^−^**/CD141**^−^**/CD1c^+^/CD5^+^/ CD11b^+^	PMID: 29356334, PMID: 31474513
	cDC3	CD14^±^/ CD163^±^/CD5**^−^**	PMID: 28428369; PMID: 31474513
	pDC	CD14**^−^**/CD123^+^/CD141**^−^**/CD1c**^−^**/CD5**^−^**	PMID: 29356334; PMID: 28167780

**TABLE 2:** BMC panel markers. Cell-surface markers used to gate and identify indicated cell types in BMC samples

Major cell population	Subset	Cell-surface markers	References
**Total T Cells**		**CD45^+^/CD3^+^/CD34^−^**	**PMID: 28069807;** **PMID: 32830910**
**CD8^+^ T Cells**		**CD45^+^/CD3^+^/CD34^−^/TCRαβ^+^/TCRγδ^−^/CD56^−^/ CD4^−^/CD8^+^**	**PMID: 28069807**
	Effector CD8	CD45RA**^−^**/CD197**^−^**	PMID: 28069807; PMID: 32830910
	Central memory CD8	CD45RA**^−^**/CD197^+^	PMID: 28069807; PMID: 32830910
	Naïve CD8	CD45RA^+^/CD197^+^	PMID: 28069807; PMID: 32830910
	TEMRA CD8	CD45RA^+^/CD197**^−^**	PMID: 10537110; PMID: 28069807; PMID: 18657274
**CD4^+^ T Cells**		**CD45^+^/CD3^+^/CD34^−^/TCRαβ^+^/TCRγδ^−^/CD56^−^/ CD4^+^/CD8^−^**	**PMID: 28069807**
	Effector CD4	CD45RA**^−^**/CD197**^−^**	PMID: 28069807; PMID: 32830910
	Central memory CD4	CD45RA**^−^**/CD197^+^	PMID: 28069807; PMID: 32830910
	Naïve CD4	CD45RA^+^/CD197^+^	PMID: 28069807; PMID: 32830910
	TEMRA CD4	CD45RA^+^/CD197**^−^**	PMID: 10537110; PMID: 28069807; PMID: 18657274
	Regulatory T cells	CD25^+^/CD127**^−^**	PMID: 28069807; PMID: 32830910
**NKT Cell**		**CD45^+^/CD3^+^/CD34^−^/TCRαβ^+^/TCRγδ^−^/CD56^+^**	**PMID: 28069807;** **PMID: 32830910**
	NKT CD8^+^	CD8^+^	PMID: 28069807
	NKT CD8-	CD8**^−^**	PMID: 28069807
**Gamma delta T Cells**		**CD45^+^/CD3^+^/CD34^−^/TCRγδ^+^**	**PMID: 32830910**
**Hematopoietic stem cells (HSC)**		**CD45^+^/CD34^+^/CD3^−^/CD38^−^/CD45RA^−^/CD90^+^**	**PMID: 24388174;** **PMID: 23708252**
**Multipotent progenitor cells (MPP)**		**CD45^+^/CD34^+^/CD3^−^/CD38^−^/CD45RA^−^/CD90^−^**	**PMID: 24388174;** **PMID: 23708252**
**Multilymphoid progenitor cells (MLP)**		**CD45^+^/CD34^+^/CD3^−^/CD38^−^/CD45RA^+^/CD90^−^**	**PMID: 24388174;** **PMID: 23708252**
	MLP I	CD10**^−^**	PMID: 24388174; PMID: 23708252
	MLP II	CD10^+^	PMID: 24388174; PMID: 23708252
**Common myeloid progenitor cells (CMP)**		**CD45^+^/CD34^+^/CD3^−^/CD38^+^/CD10^−^/ CD45RA^−^/CD135^+^**	**PMID: 24388174;** **PMID: 23708252**
**Granulocyte-monocyte progenitor cells (GMP)**		**CD45^+^/CD34^+^/CD3^−^/CD38^+^/CD10^−^/ CD45RA^+^/CD135^+^**	**PMID: 24388174;** **PMID: 23708252**
**Megakaryocyte-erythroid progenitor cells (MEP)**		**CD45^+^/CD34^+^/CD3^−^/CD38^+^/CD10^−^/ CD45RA^−^/CD135^−^**	**PMID: 24388174;** **PMID: 23708252**
**CD10^+^ Progenitors**		**CD45^+^/CD34^+^/CD3^−^/CD38^+^/CD10^+^**	**PMID: 24388174;** **PMID: 23708252**
**B cells**		**CD45^+^/CD3^−^/CD34^−^/CD20^+^/CD19^+^**	**PMID: 28069807;** **PMID: 32830910**
	Plasmablasts	CD20^−^/CD38^+^	PMID: 32830910
**NK cells**		**CD45^+^/CD3^−^/CD34^−^/CD20^−^/CD19^−^**	**PMID: 28069807;** **PMID: 32830910**
	Early NK	CD56^+^/CD16**^−^**	PMID: 32830910
	Mature NK	CD56^+^/CD16^+^	PMID: 32830910
	Terminal NK	CD16^+^/CD56**^−^**	PMID: 23650273; PMID: 32830910
**Dendritic cells**		**CD45^+^/CD3^−^/CD34^−^/CD20^−^/CD19^−^/HLA-DR^+^/ CD14^−^**	**PMID: 32830910**
	pDC	CD11c**^−^**/CD123^+^	PMID: 32830910
	CD16^+^ cDC	CD11c^+^/CD123**^−^**/CD16^+^	PMID: 31749181
	CD16- cDC	CD11c^+^/CD123**^−^**/CD16**^−^**	PMID: 31749181
**Innate lymphoid cells (ILC)**		**CD45^+^/CD3^−^/CD34^−^/CD20^−^/CD19^−^/HLA-DR^−^/ CD14^−^/CD123^−^/CD127^+^**	**PMID: 32830910**
**Nonclassical monocytes**		**CD45^+^/CD3^−^/CD34^−^/CD20^−^/CD19^−^/CD14^−^/ CD16^+^**	**PMID: 32830910;** **PMID: 30684003**
**Classical monocytes**		**CD45^+^/CD3^−^/CD34^−^/CD20^−^/CD19^−^/CD14^+^/ CD16^−^**	**PMID: 32830910;** **PMID: 30684003**
**MDSC-like**		**CD45^+^/CD3^−^/CD34^−^/CD20^−^/CD19^−^/HLA-DR^−^/ CD14^+^/CD11b^+^/CD33^+^**	**PMID: 30684003**

### PBMC Isolation and Storage

Blood samples were collected in cell preparation tubes (CPT; Becton Dickinson) allowing for collection of PBMCs. PBMCs were isolated via Ficoll-density gradient centrifugation within 2 hours of blood collection. Tubes were first inverted eight to 10 times followed by centrifugation for 30 minutes at 1,600 × *g* at room temperature with acceleration and brake set to the lowest setting. Tubes were inverted eight to 10 times to resuspend PBMCs in plasma. The plasma and PBMC suspensions were pooled into a 50 mL conical tube and centrifuged for 10 minutes at 900 × *g* at room temperature with acceleration and breaks set to 9. Excess supernatant was aspirated without disturbing the PBMC pellet. PBMCs were washed in 20 mL of wash media [1% heat-inactivated FBS (Thermo Fisher Scientific) in PBS (Life Technologies)] and counted using a Countess III automated cell counter (Thermo Fisher Scientific). Tubes were next centrifuged for 10 minutes at 900 × *g* at room temperature with brakes and acceleration set to 9 to remove the wash media. PBMCs were viably frozen in 1 mL of freezing media [90% heat-inactivated FBS, 10% DMSO (Sigma-Aldrich)] per 5 × 10^6^ cells. PBMCs were cryopreserved in liquid nitrogen until needed for analyses.

### PBMC Thawing

Cryopreserved PBMCs were thawed at 37°C before staining. Briefly, PBMC-containing cryovials were held on the surface of the water bath for approximately 1 minute until only a sliver of ice remained. A total of 1 mL of warm RPMI media (Thermo Fisher Scientific) was then added into the cryovial dropwise over a 40-second period. Cell suspensions were then transferred to 50-mL conical tubes containing 8 mL of warm RPMI media. In cases where multiple cryovials from the same donor were thawed, vials were combined in the same conical tube. Cells were centrifuged for 10 minutes 1,000 × *g* at room temperature. Supernatant was aspirated and the pellet was resuspended in the desired volume of warm RPMI media. Cell number and viability were determined with a hemacytometer using the Trypan Blue method and further validated by the Cytek Aurora SpectroFlo software.

### BMC Isolation and Preparation

Commercially available BMCs (Lonza, catalog no. 2M-125C) were purchased as cryopreserved vials. Lonza isolated cells from whole bone marrow aspirate samples using density gradient separation. The isolated cells are received suspended in Hank's Balance Salt Solution with 5 mmol/L ethylenediaminetetraacetic acid and 0.5% BSA. BMCs were cryopreserved in liquid nitrogen until needed. On February 2, 2022, March 28, 2022, and July 14, 2022, BMCs were thawed in a 37°C water bath, pelleted, and resuspended in protein-free PBS with FBS prior to antibody labeling and viability staining. A total of 0.2–2.0 × 10^6^ cells were tested using the BMC antibody panel.

### Antibody Titration Testing

All antibodies were diluted in a final volume of 200 µL of staining buffer (BD Stain Buffer, RRID: AB_2869007) except for the viability dye, which was diluted in PBS (Life Technologies). All antibodies were tested in a 4-fold serial dilution starting with the manufacturer's recommendation as the highest dilution. Titration performance was assessed by first examining the spectral signature of the fluorochrome compared with the expected signature. Histogram plots were then used to evaluate the separation between the positive and negative events at the highest peak channel. Stain index was also assessed to further confirm titration accuracy. Further titration was performed until an optimal dilution was achieved (Supplementary Tables S1 and S2). Both beads and cells were tested as reference controls for several of the fluorochromes, but we noticed slight unmixing errors when using beads. Therefore, for all work in this study, cells were used as the reference controls to evaluate panel performance. Titrations for human PBMC antibodies from Cytek Biosciences and titrations for all BMC antibodies (Supplementary Table S3) were performed by Cytek Biosciences.

### Unmixing Accuracy and Marker Performance Assessment

Data were unmixed using the Cytek Aurora SpectroFlo software v2.2 unmixing algorithm. Per cytometer guidelines, autofluorescence extraction was employed in the analysis of all panels to remove inherent baseline autofluorescence exhibited by the cells, to improve fidelity of results. Unmixing accuracy was assessed by screening NxN plot permutations for each marker against each other marker. In addition, single-color and multicolor histogram overlays for each marker were generated using the FlowJo v9 software to compare the resolution for each marker. One marker, TCRαβ-BV510, in the PBMC panel was observed to have a significantly dimmer resolution in the multicolor sample than as a single-color control. To improve TCRαβ-BV510 resolution, we doubled the antibody titer and tested a 10-minute preincubation before adding the remaining antibodies in the cocktail. Doubling the titer improved the accuracy and reliability of our data. No action was taken in cases where the marker resolution was identical or whether there was a slight spread that did not negatively impact the overall performance of the panel.

To further confirm accurate performance of markers with continuous staining or low expression, we employed a robust gating strategy based on unlabeled and single-color controls. The use of these controls enables one to assess the staining characteristics in the absence of fluorochrome and with individual fluorochromes, and subsequently enables one to establish gates for each marker. In addition, we confirmed the spectral flow signature for each marker to ensure it was similar to the expected signature and also visually inspected the gate positions for each marker across all samples to ensure consistency and reliability of results. Further validation was performed through visual screening of the NxN plots for each marker. To enhance the accuracy of the analysis for markers with low cell-surface expression or cells of low abundance, we recommend staining a higher number of cells (at least 2 × 10^6^). This approach allows for accurate gating of positive and negative events.

### Staining Protocol

A total of 2.5 × 10^5^ PBMCs were used to generate single-color control tests and 2 × 10^6^ cells were used for each multicolor experimental stain. All staining reactions were performed in FACS tubes. Tubes were centrifuged at 1,000 × *g* for 5 minutes at room temperature to remove the RPMI media. Cell pellet was washed in 200 µL PBS. Next, 100 µL of ViaDye Red Viability dye was added to the viability single color control and sample tubes and cells were stained for 20 minutes. A total of 100 µL of staining buffer was added to the other single color control and unlabeled tubes. After the 20-minute incubation, tubes were centrifuged and supernatant was aspirated. Cells were then washed in 200 µL staining buffer, centrifuged, and supernatant aspirated. Next, blocking was performed using a cocktail of 5 µL True-Stain Monocyte Blocker (BioLegend) and 5 µL Human TruStain FcX (BioLegend) for 20 minutes at room temperature. Cells were centrifuged and supernatant aspirated. Blocked cells were then stained for 30 minutes at room temperature with single stains for the single color control tubes or the full cocktail of antibodies (including 10 µL Brilliant Staining Buffer Plus [BD Biosciences, RRID:AB_2869761)] in a 200 µL volume for the sample tubes—antibodies should be centrifuged prior to use to minimize the risk of antibody aggregates which can impact data fidelity. A total of 100 µL of staining buffer was added to the unlabeled tube. Cells were centrifuged to remove the antibody followed by a wash in 200 µL of staining buffer. Next, cells were fixed in 1% paraformaldehyde (PFA) for 20 minutes at room temperature. A final wash was performed to remove extra PFA. Cells were then resuspended in 300 µL of staining buffer and tubes run on the Cytek Aurora on the same day. All staining steps were performed at room temperature in the dark. All centrifugation steps were performed at 1,000 × *g* for 5 minutes. Similar steps were followed by Cytek Biosciences for the BMC panel.

### Quantification and Statistical Analyses

Unmixed data were imported into the OMIQ software (app.omiq.ai) for analysis. Manual gating was performed using established gating strategies. Heat maps and Uniform Manifold Approximation and Projection (UMAP) dimensionality reduction analysis were performed using OMIQ and the R package Seurat. To account for BMC batch effects using Harmony integration (20), raw fluorescence values for each viable cell in each channel were exported for each BMC sample from OMIQ flow cytometry software as a .csv file. The files were then read into R and a merged Seurat object of all three samples was created with every fluorescence channel except for viability and autofluorescence as features for each individual cell. The Seurat object's counts data, which contained these raw fluorescence values for each cell, was moved to the “data” slot, and the standard Seurat pipeline was applied with Harmony to generate integrated UMAP coordinates. This pipeline includes scaling data to account for high discrepancies in numerical values for each fluorescence channel, such that the range of values for each channel was centered around 0 with a range from −1 to 1. Next, an elbow plot determined the necessity for 20 principal components to be implemented in Harmony integration. The object was then integrated using Harmony, and a UMAP was generated from the results of that integration. UMAP coordinates were saved for each cell, and these coordinates were added to the data structure including all channel values as additional data columns per cell. The data frame was then written into a .fcs file using the “flowFrame” and “write.FCS” functions in the flowCore R package (21). The resulting .fcs files were reuploaded to OMIQ for subsequent analysis. To objectively determine the need for application of Harmony, we included an assessment of local inverse Simpson indices (ref. 20; LISI score) for each cell in every UMAP using the “compute_lisi" function in the lisi R package following documentation available on the developer's GitHub (20). For UMAPs with three samples (and thus a maximum LISI score of 3), we defined high LISI cells as those with a score >2. For UMAPs with two samples (and thus a maximum LISI score of 2), we defined high LISI cells as those with a score >1.667.

### Custom Antibodies

Upon initiation, this study included nine custom antibody/fluorophore conjugates developed and validated by Cytek Biosciences for the PBMC panels: CD45-cFluorB548, CD123-cFluorV450, CD163-cFluorV610, CD314-cFluorR685, CD14-cFluorR720, CD20-cFluorB675, CD38-cFlourR720, CD4-cFlourYG584, and ViaDye Red viability stain. In addition, the study included 16 custom antibody/fluorophore conjugates developed and validated by Cytek Biosciences for the BMC panel: CD123-cFluorV450, CD14-cFluorV547, CD45-cFluorB548, CD16-cFluorB677, CD20-BB755, CD25-cFluorBYG575, CD90-cFluorBYG610, CD56-cFluorYG610, TCRg/d-cFluorBYG710, CD163-cFluorBYG750, CD197-cFluorBYG781, CD33-cFluorR659, CD45RA-cFluorR685, HLA-DR-cFluorR720, CD10-cFluorR780, CD38-cFluorR840. Four of these antibodies [CD38-cFlourR720, CD4-cFlourYG584 ,ViaDye Red and CD45-cFluorB548 (BMC panel)] are now commercially available for purchase through Cytek Biosciences.

### Data and Code Availability

Flow cytometry data are available on Figshare (DOI: 10.6084/m9.figshare.25057739). Source code for the Harmony implementation within the Seurat framework is available at https://github.com/adrienneparsons/SpectralFlow23. Further information and reasonable requests should be directed to the lead contact, Sandra S. McAllister (smcallister1@bwh.harvard.edu).

## Results

### Design of Spectral Flow Cytometry Immunophenotyping Panels

We designed three panels to perform deep immunophenotyping of human blood and bone marrow cells on a four-laser spectral flow cytometer (violet—405 nm, blue—488 nm, yellow/green—561 nm, and red—640 nm). The PBMCs are profiled using two panels. First, a 27-marker T/B panel characterizes CD8^+^ and CD4^+^ T cells, γδT cells, NKT cells, NK cells, and B cells, including their various functional subsets and checkpoint proteins (Table 1; Supplementary Table S1). A second 25-marker M/N/D panel characterizes monocytes, NK cells, and dendritic cells, including their various functional subsets (Table 1; Supplementary Table S2). A 32-marker human BMC panel profiles hematopoietic stem cells (HSCs), various progenitor populations, CD8^+^ and CD4^+^ T cells, γδT cells, NKT cells, NK cells, B cells, monocytic and myeloid cells, and DCs, including various relevant functional subsets (Table 2; Supplementary Table S3).

### Specificity and Performance of Antibody-fluorophore Panels

We wanted to ensure optimal performance of the panels and avoid cross-reactivity between different antibody-fluorophore conjugates. For these analyses, we used cryopreserved PBMCs obtained from women who were seen at the Brigham and Women's Hospital B-PREP clinic (22). We confirmed cell viability upon thawing using the trypan blue method as well as upon data analysis using the Cytek Aurora SpectroFlo software. These methods consistently revealed a cell viability of greater than 95% for the cryopreserved PBMCs (Supplementary Table S4).

We compared the performance of each antibody-fluorophore conjugate alone as a single stain to when it was included in the complete cocktail for each panel to determine whether an optimal resolution was maintained. We visually confirmed the expected spectral signature for each fluorochrome as a single stain and as a component of its respective multi-antibody panel. We next generated single-color and multicolor histogram overlays to visualize the results of the antibody performance. For the PBMC panels, the histograms aligned between the single stain and the multicolor cocktail as indicated by agreement of the magnitude and spread of the positive and negative peaks (Supplementary Fig. S1A and S1B; ref. 18). No action was taken in the few cases where there was minimal spread for any given marker that did not impact the overall panel performance (Supplementary Fig. S1A and S1B). Users are encouraged to exercise discretion in determining acceptable levels of spread for the purposes of their experimentation.

To ensure that markers could be clearly differentiated from one another, we visualized density plots for each marker against all other markers in each panel and generated multiplot panels (NxN plots; Supplementary Fig. S2A, S2B, S2E, and S2F). One of the benefits of full spectrum flow cytometry is that it limits the requirement for manual compensation of emission spectra overlap (5, 19). Instead, multispectral flow cytometry relies on an ordinary least squares unmixing algorithm to distinguish fluorophore signatures by calculating the full spectral signature for each fluorophore across all detectors (5, 19). In addition, unmixing accuracy is improved by the cellular autofluorescence extraction tool, which removes inherent baseline autofluorescence exhibited by the cells and is recommended by the cytometer manufacturer (5, 19). Nevertheless, additional manual compensation can still be applied to spectral data after unmixing when necessary. In this case, after manually examining each of the NxN plots, we noticed a signal spillover from BYG575 (conjugated to CD159c) and BYG710 (conjugated to CD19) in the M/N/D panel, indicating an unmixing error. On the basis of the misalignment of the negative and positive populations observed in the density plot, we determined that additional compensation was required to resolve this interaction (Supplementary Fig. S2C). We therefore applied a minimal correction of −2.79 from BYG575 into BYG710 using the Cytek Aurora SpectroFlo software compensation tool and reassessed the NxN plots for any changes in the overall panel performance (Supplementary Fig. S2D).

In the BMC panel, aberrant spillover was observed in every NxN association for the NovaFluor Blue 610-70S—CD19 antibody (Supplementary Fig. S2E). However, this spillover was also observed for this antibody in the single-color control before unmixing. We therefore concluded that this particular antibody was subject to aggregation and that the spillover was not the consequence of error in unmixing or panel design. Aggregates such as this can be removed by antibody centrifugation prior to staining the cells as well as using a gating strategy to remove data resulting from aggregation (Supplementary Fig. S2G). After these corrections, we were satisfied that distinct cell populations could be clearly and accurately differentiated in both PBMC panels (Supplementary Fig. S2A and S2B) and the BMC panel (Supplementary Fig. S2F).

Three antibodies are used in both the T/B and the M/N/D panels—CD45-B548, CD3-R840, and HLA-DR-R780 – thus providing an opportunity to assure staining and performance consistency between panels. The histograms representing the staining for each antibody showed concordance between panels (Supplementary Fig. S3A). We then calculated the stain index for each antibody in three donor PBMC samples in both panels using the formula:



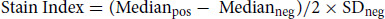



whereby “Median” is the median fluorescence intensity (MFI) for either the positive or negative peak of each fluorophore, and “SD” is the standard deviation of MFI. For each of these three markers, there was no statistically significant difference in stain index between the panels (Supplementary Fig. S3B) by two-way ANOVA, indicating equivalent performance in each panel.

### PBMC Gating Strategies

**FIGURE 1:**
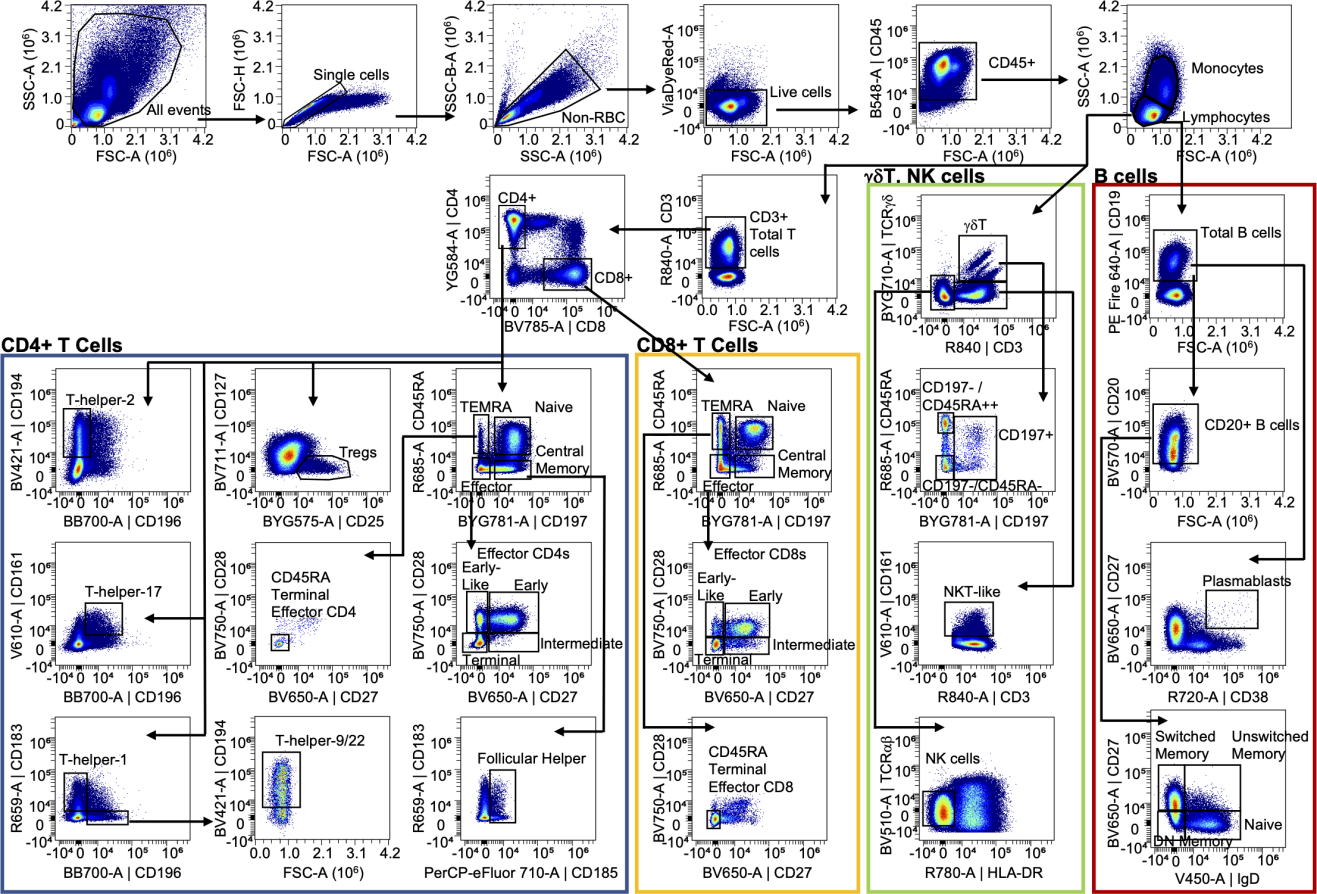
PBMC Panel 1: Gating strategy for T and B cells. Density plots of the gating strategy used to identify indicated PBMC cell types in the T/B panel. Arrows denote sequential steps in the gating strategy and are used to visualize the relationships across populations. Density plots represent 553,000 CD45^+^ live single cells from concatenation of events from all donors (*n* = 3).

**FIGURE 2:**
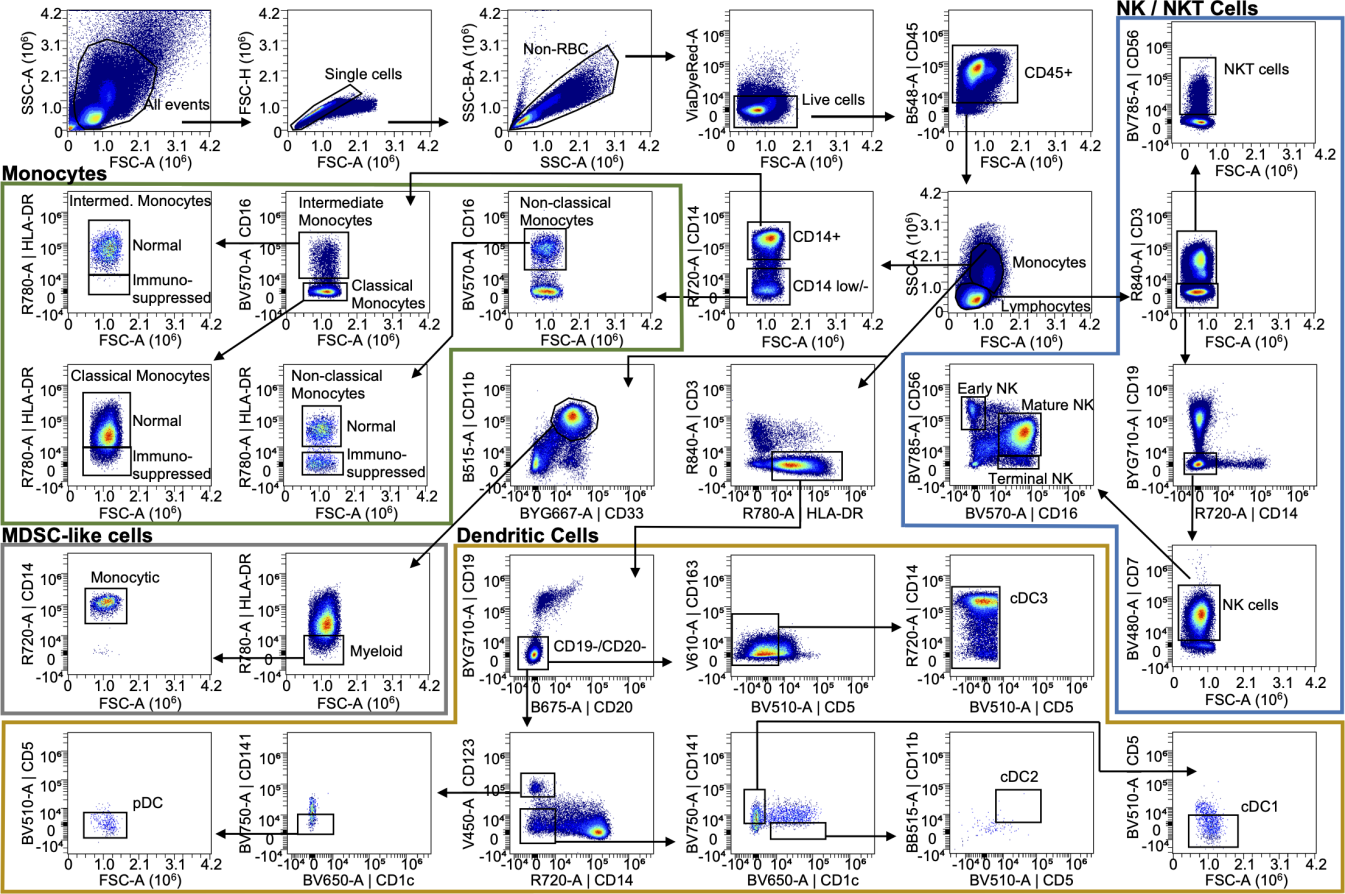
PBMC Panel 2: Gating strategy for monocyte, NK, and dendritic cells. Density plots of the gating strategy used to identify indicated PBMC cell types in the M/N/D panel. Arrows denote sequential steps in the gating strategy and are used to visualize the relationships across populations. Density plots represent 534,000 CD45^+^ live single cells from concatenation of events from all donors (*n* = 3).

Having validated panel performance, we designed gating strategies to calculate cell frequencies. For all three panels, we first captured only single cells, eliminated red blood cells, and captured live CD45-positive (CD45^+^) cells (Figs. 1 and 2). For the T/B panel, we gated lymphocytes based on size (FSC-A) and granularity (SSC-A) and used that gate for subsequent assessment of cell frequencies (Fig. 1). The cell-surface markers, CD3, CD4, and CD8 were used to identify major T-cell populations, and combinations of CD45RA and CD197 (CCR7) were used to distinguish naïve, central memory, effector memory, and terminal effector memory (TEMRA) subpopulations (Fig. 1; Table 1). CD27 and CD28 were used to further refine early, early-like, intermediate, and terminal and CD45RA terminal effector T cells (Fig. 1; Table 1).

The CD4^+^ population was further assessed for frequency of regulatory T cells (T_regs_), various Th cells, and follicular helper cells. Specifically, we used: CD127 (IL7 receptor-alpha) and CD25 (IL2 receptor-alpha) for T_regs_, CD196 and CD183 for Th1 cells, CD196 and CD194 for Th2 cells, CD196 and CD161 for Th17 cells, CD194 and CD196 for Th9/22 cells, and CD183 and CD185 for follicular helper cells (Fig. 1; Table 1).

Markers to distinguish γδT cells and NK cells were also included in the T/B panel. CD197 and CD45RA were used to identify various subsets of CD3^+^/TCRγδ^+^ T cells (Fig. 1 and Table 1). CD3^+^/TCRγδ^−^/CD161^+^ cells were identified as NKT like cells, and CD3^−^/TCRγδ^−^/TCRαβ^−^/HLA-DR^−^ cells were identified as NK cells in the T/B panel (Fig. 1; Table 1).

B cells in the T/B panel were first identified by their cell-surface expression of CD19 (Fig. 1; Table 1). From there, plasmablasts could be identified as CD38^+^/CD27^+^ cells, and various subsets of B cells [switched memory, unswitched memory, double-negative (DN) memory, and naïve] were identified using CD20, IgD, and CD27 expression (Fig. 1; Table 1).

We also included markers in the T/B panel that would enable one to quantify the frequency of activated T cells as well as expression of select checkpoint proteins on various cell populations. As an example, we used cells identified in the CD3^+^ gate and represented activated T cells as CD38^+^/HLA-DR^+^ (Supplementary Fig. S4). Checkpoint proteins PD1 (CD279), Tim3 (CD366), Lag3 (CD223), and CTLA4 (CD152) were also expressed on the CD3^+^ cells (Supplementary Fig. S4).

For the M/N/D panel, we distinguished monocytes from lymphocytes based on size (FSC-A) and granularity (SSC-A) and used those gates for subsequent assessment of cell frequencies (Fig. 2). From the monocyte size gate, CD14 was used to distinguish classical and intermediate monocytes from nonclassical monocytes (Fig. 2; Table 1). Expression of CD16^+^ and HLA-DR were then used to identify various subsets of those monocyte populations (Fig. 2; Table 1). Immunosuppressive monocytes were identified as CD11b^+^/CD33^+^ and then classified as immune suppressive myeloid (HLA-DR^−^) or monocytic (CD14^+^) cells (Fig. 2; Table 1).

Various DCs were identified in the monocyte size gate by first selecting a CD3^−^/HLA-DR^+^/CD19^−^/CD20^−^ population (Fig. 2; Table 1). From there, plasmacytoid dendritic cells (pDCs) were identified as CD14^−^/CD123^+^/CD141^−^/CD1c^−^/CD5^−^ (Fig. 2; Table 1). The CD14^−^/CD123^−^ cells were further characterized by their expression of CD1c and CD141, whereby CD1c^−^/CD141^+^/CD5^−^ were identified as cDC1 cells and the CD1c^+^/CD141^−^/CD5^+^/CD11b^+^ cells were cDC2 cells (Fig. 2; Table 1). From the CD19^−^/CD20^−^ gate, cDC3 cells were identified as CD163^+^/^−^/CD5^−^/CD14^+/−^ (Fig. 2; Table 1).

Further assessment of the lymphocyte size gate (FSC-A vs. SSC-A) enabled us to identify NK and NKT cells. The CD3^+^/CD56^+^ cells were identified as NKT cells (Fig. 2; Table 1). The CD3^−^/CD14^−^/CD19^−^/CD7^+^ population was identified as NK cells, and assessment of CD16 and CD56 enabled identification of early (CD56^+^/CD16^−^), mature (CD56^+^/CD16^+^), and terminal (CD56^−^/CD16^+^) NK cell subsets (Fig. 2; Table 1). The M/N/D panel also includes a variety of NK cell functional markers, including CD94 (KLRD1), CD314 (NKG2D), CD159c (NKG2C), CD337 (NKp30), CD335 (NKp46), CD336 (NKp44), CD158 (KIR2DL1/S1/S3/S5), CD366 (TIM3; Supplementary Fig. S5).

We validated the consistency between the two PBMC panels by examining the lymphocyte:CD45 ratio in the three patient samples in each panel. Moreover, because NK cells were identified in both panels using slightly different gating strategies, we were able to assess the agreement between panels by calculating the NK:CD45 ratio for each sample in each panel. For each sample, our gating strategies (Figs. 1 and 2) were used to quantify the number of lymphocytes, CD45^+^ cells, and NK cells for both the T/B and M/N/D panels. The ratio of lymphocytes:CD45^+^ cells and NK:CD45^+^ cells was then calculated. A two-way ANOVA revealed no statistical difference due to sample or panel used for either lymphocyte:CD45 ratio or NK:CD45 ratio (Supplementary Fig. S3C).

All cell types of interest across both PBMC panels, including rare populations, could be detected with an input of 2 × 10^6^ cells. We had collected donor blood samples in standard 8-mL CPT, which yield a minimum of 6 × 10^6^ cells per tube. Therefore, our two-panel PBMC pipeline requires only a portion of the donor sample, thereby preserving sufficient cells for other analyses.

### BMC Gating Strategy

**FIGURE 3:**
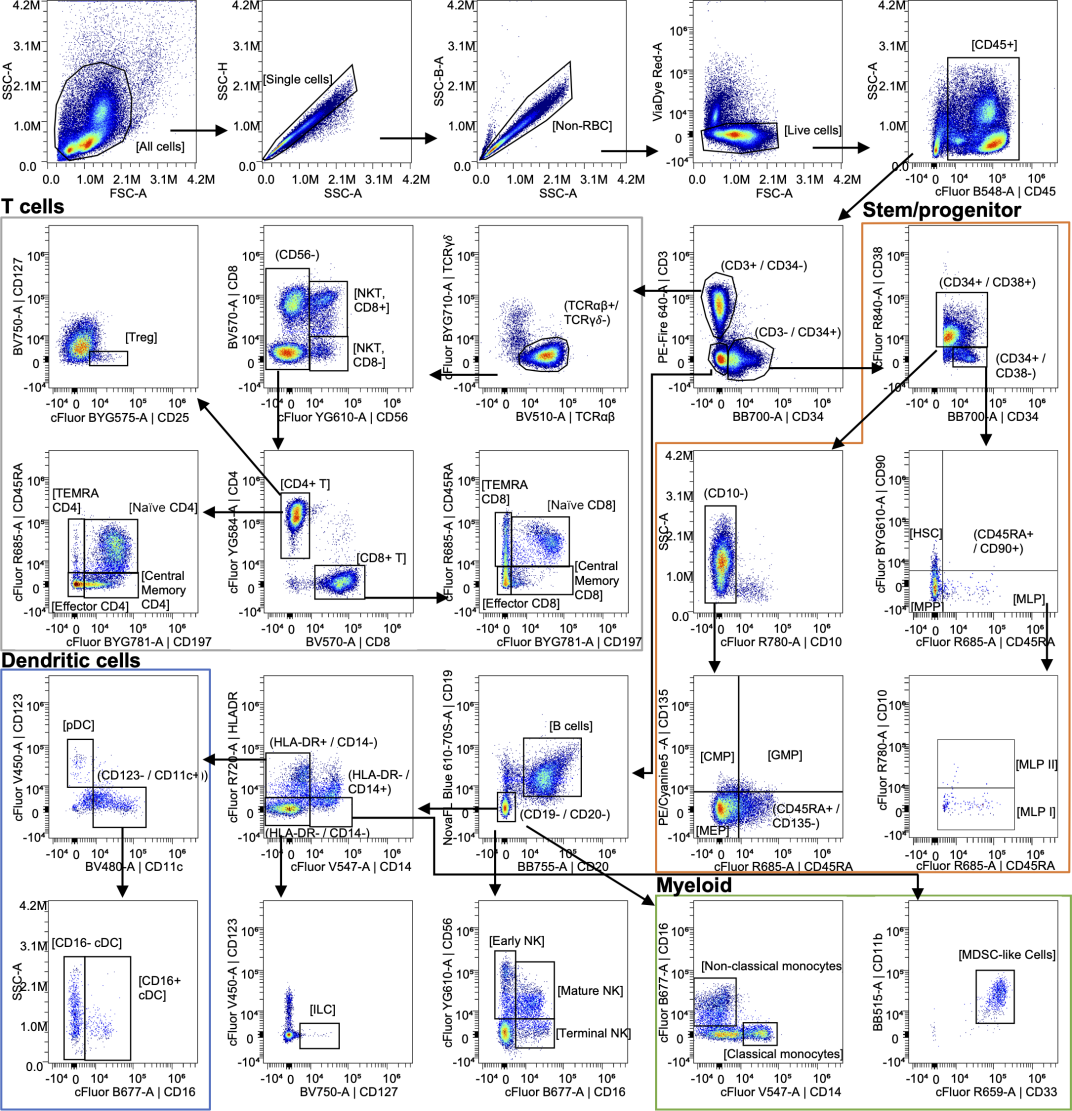
BMC panel gating strategy. Density plots of the gating strategy used to identify indicated BMC cell types. Arrows denote sequential steps in the gating strategy and are used to visualize the relationships across populations. Canonically gated populations are indicated with brackets, while intermediate steps are indicated with parentheses. Data presented are derived from one representative sample and represent 72,000 CD45^+^ live single cells.

Bone marrow cells were first gated to exclude debris and doublets using FSC-A and SSC-A (Fig. 3). Viable cells were selected as ViaDye Red-negative. CD45^+^ blood cells were then selected and separated into CD3^+^/CD34^−^ T lymphocytes, CD3^−^/CD34^+^ progenitors, and CD3^−^/CD34^−^ cells. The CD3^+^/CD34^−^ T lymphocytes were subsequently gated as TCRγδ^−^/TCRαβ^+^. From the TCRαβ^+^ population, CD56^+^ NKT cells were determined to be either CD8^+^ or CD8^−^ (Fig. 3; Table 2). All CD56^−^ T lymphocytes were gated as CD4^+^ or CD8^+^, and those two populations were subcategorized as either naïve (CD197^+^/CD45RA^+^), effector (CD197^−^/CD45RA-), central memory (CD197^+^/CD45RA^−^), or TEMRA (CD197^−^/CD45RA^+^; Fig. 3; Table 2). T_regs_ were identified within the CD4^+^ T lymphocyte population as CD25^+^/CD127^−^ (Fig. 3; Table 2). Additional markers within the panel include identifiers of immunosuppression (CD274, CD366, CD163, CD206) and antigen presentation (CD74, CD80, CD86; Supplementary Table S2).

Within CD3^−^/CD34^+^ cells, HSCs reside within the CD34^+^/CD38^−^ population and downstream progenitors within the CD34^+^/CD38^+^ population. The CD34^+^/CD38^−^ cells were subcategorized as HSCs (CD45RA^−^/CD90^+^), multipotent progenitors (MPP; CD45RA^−^/CD90^−^), and multilymphoid progenitors (MLP; CD45RA^+^/CD90^−^; Fig. 3; Table 2). MLPs were further classified as MLP I (CD10^−^) or MLP II (CD10^+^; Fig. 3; Table 2). The CD34^+^/CD38^+^ cells were subsequently gated as CD10^−^ and the resulting populations were identified as common myeloid progenitors (CMP; CD45RA^−^/CD135^+^), granulocyte-monocyte progenitors (GMP; CD45RA^+^/CD135^+^), or megakaryocyte-erythroid progenitors (MEP; CD45RA^−^/CD135^−^; Fig. 3; Table 2).

B lymphocytes were identified from the CD3^−^/CD34^−^ population as CD19^+^/CD20^+^ (Fig. 3; Table 2). The CD19^−^/CD20^−^ cells from the CD3^−^/CD34^−^ parent gate were then classified on the basis of cell-surface expression of CD14, CD16, CD56, and/or HLA-DR. Specifically, CD14 and CD16 identified classical monocytes (CD14^+^/CD16^−^) and nonclassical monocytes (CD14^−^/CD16^+^; Fig. 3; Table 2). NK cell subpopulations were identified as early NK (CD16^−^/CD56^+^), mature NK (CD16^+^/CD56^+^), or terminal NK (CD16^+^/CD56^−^; Fig. 3; Table 2). Finally, the CD19^−^/CD20^−^ parent gate was subdivided into populations based on levels of CD14 and HLA-DR, with innate lymphoid cells (ILCs) identified as CD127^+^/CD123^−^ from the CD14^−^/HLA-DR^−^ population, myeloid-derived suppressor cell-like cells being identified as CD33^+^/CD11b^+^ from the CD14^+^/HLA-DR^−^ population, and DCs being identified from the CD14^−^/HLA-DR^+^ population (Fig. 3; Table 2). DCs were subclassified as either cDCs (CD123^−^/CD11c^+^) or pDCs (CD123^+^/CD11c^−^; Fig. 3; Table 2). Two populations of cDCs were identified as CD16^+^ cDCs and CD16^−^ cDCs (Fig. 3; Table 2).

### Cell Frequencies, Cell Relationships, and Identifying Rare or New Populations

Cell frequencies of interest can be calculated as percentages of their antecedent gates in two-dimensional flow plots using standard flow cytometry methods [e.g., parent gates (Figs. 1, 2, and 3)]. Spectral flow cytometry data are also amenable to analyses that fully leverage the high number of parameters that can be simultaneously measured. Such high-dimensional data require special considerations. For example, it would be incorrect to represent a sample as an added composite of each of the defined cell types, because some parent gates are based on a single marker rather than a defined cell type and certain cell types are identified via negative selection. Hence, we sought visualization methods that avoid misrepresentations and that are suitable for high-dimensional data.

**FIGURE 4:**
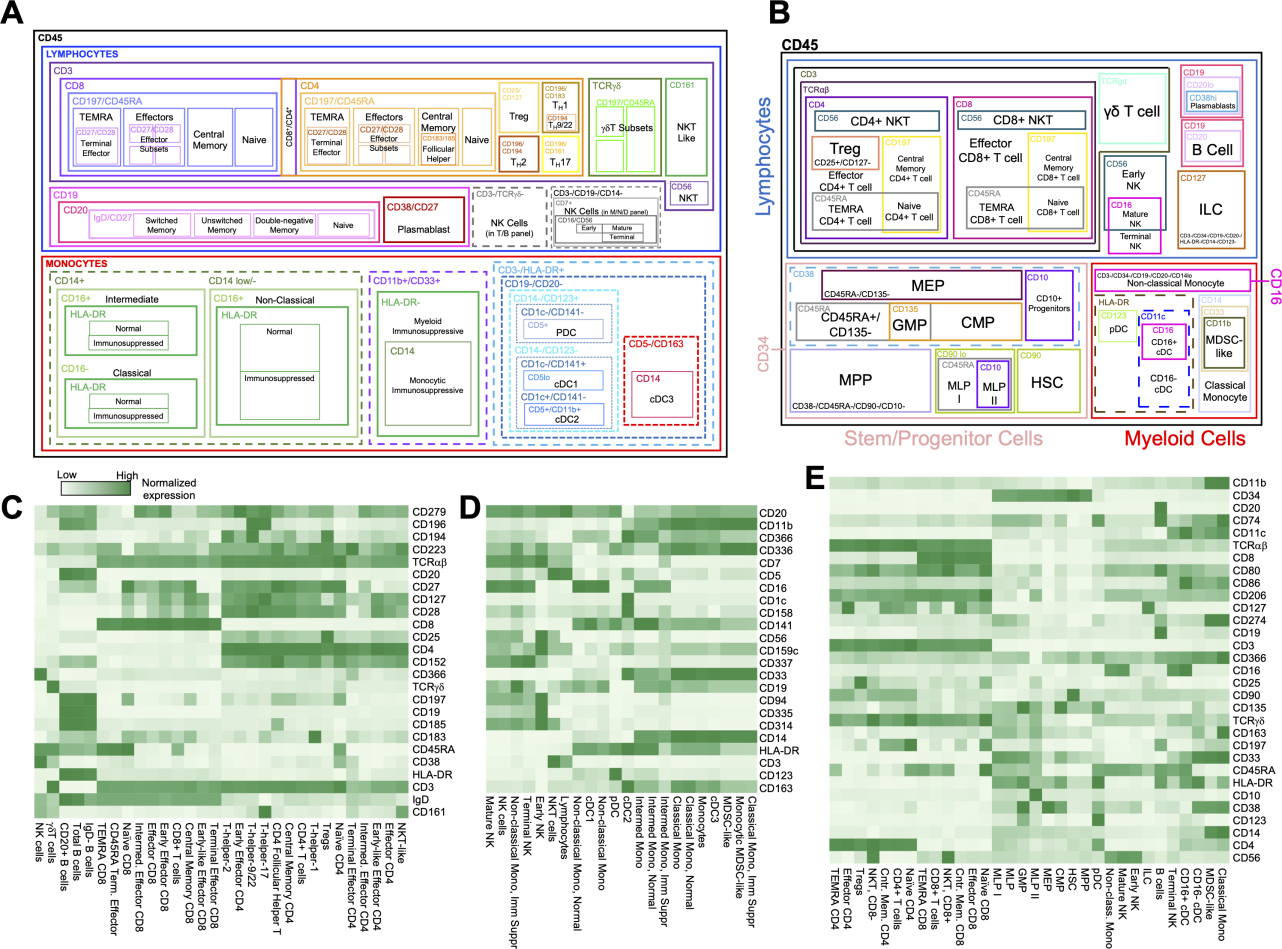
Cell relationships and marker complexity. **A,** PBMC cell relationship schematic. **B,** BMC cell relationship schematic. Solid lines in A and B reflect canonically gated cell populations, while dashed lines represent intermediate gating steps. **C**–**E,** Clustered heatmaps displaying relative expression of different markers across defined cell types depicted in A and B. Columns represents the expression of each marker while rows indicate the defined cell types. The marker mean fluorescence intensity is displayed on a scale from white (lowest) to green (highest).

To begin, we created schematics to visualize the complexity and cell relationships in the PBMC (Fig. 4A) and BMC (Fig. 4B) panels. Next, we generated heat maps to visualize the relative expression of different markers across the various defined cell populations; this approach aids in visualization of complex multiparametric data and underscores the reliability of our gating strategy by visually demonstrating the expected expression patterns of key markers on distinct cell populations. The expected markers labeled cell types defined by our three gating strategies, and unsupervised clustering confirmed the relationship between cell types in our datasets (Fig. 4C–E). For example, CD3 is expressed on T cells and is absent from B cells, while only bone marrow progenitor populations are positive for CD34. Furthermore, rare populations, such as cDC2s and HSCs, display expected marker trends despite limited numbers of cells in our samples. Similar visualization using heat maps such as these can also be particularly useful for studying heterogenous cell populations and provide an intuitive way to explore large datasets and identify trends that may not be immediately apparent from numerical data.

Our pipelines also enable the identification of rare or unexpected populations of interest. For example, CD4^+^/CD8^+^ double-positive (DP) cells in the periphery are not well understood (23–25). These cells have been reported to be involved in a variety of pathologic conditions, including autoimmune diseases and cancer; however, their functionality remains largely unclear (23, 25). We observed distinct CD4hi/CD8low and CD4low/CD8hi DP populations (Supplementary Fig. S6A). Basophils, which are estimated to represent 0.1%–0.5% of cells in PBMC preparations, were detectable in our dataset as CD45^int^/CD123^hi^/HLA-DR^−^ cells (Supplementary Fig. S6B; refs. 18, 26). As a final example, the presence of ILCs can be inferred in our T/B panel from gating strategy: CD45^+^/CD3^−^/CD8^−^/CD4^−^/TCRγδ^−^/CD19^−^/CD20^−^/HLA-DR^−^/CD127^+^ (Supplementary Fig. S6C; ref. 27). Hence, our panels can reveal information about rare and as-yet undefined cell populations.

### Patient-specific Immune Profiles and Detection of Malignancy

**FIGURE 5:**
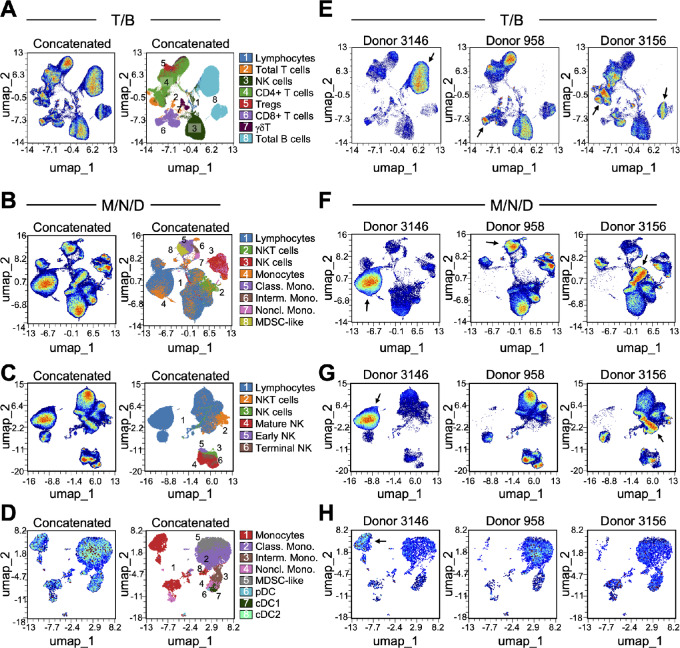
Dimensionality reduction to detect differences between PBMC donors. **A–D,** UMAP coordinates generated from concatenated data of live, non-red blood cell singlets and 85,000 lymphocytes (**A**), 100,000 CD45^+^ cells (**B**), 90,000 lymphocytes (**C**) or 5,000 monocytes (**D**) from each donor (*n* = 3). Plots on the left indicate clustering of cells in two-dimensional space; plots on the right show canonically gated major cell populations overlaid onto each UMAP. **E–H,** UMAP for each of 3 individual donors. T/B panel UMAPs (A and E); M/N/D panel total CD45^+^ cells UMAPs (B and F); M/N/D panel lymphocytes UMAPs (C and G); M/N/D panel monocytes UMAPs (D and H). Arrows (in E, F, G, and H) indicate examples of cell populations that differ in abundance between donor samples.

A relatively new visualization strategy that has gained traction for high-dimensional single-cell sequencing data is dimensionality reduction by UMAP (28). To test whether this technique could meaningfully represent the intricate relationships between markers in our spectral flow cytometry data without the constraints of conventional two-dimensional gating strategies, we generated UMAPs for the T/B panel depicting concatenated donor data based on the CD45^+^ lymphocyte population (Fig 5A). For the M/N/D panel, we visualized concatenated donor data for the entire CD45^+^ population (Fig. 5B), the CD45^+^ lymphocytes (Fig. 5C), and the CD45^+^ monocyte populations separately (Fig. 5D).

In the UMAP visualizations, cells with similar underlying fluorescence values are positioned closer together (Fig. 5). To further examine this, we scrutinized the markers associated with UMAP coordinates by coloring the UMAPs according to expression intensity of each marker (Supplementary Fig. S7). This approach enabled us to visualize the fluorescence intensity of each marker on each cell to determine which markers are present on distinct clusters on the UMAP, and to characterize how each marker is distributed across all cells. For example, in the T/B panel, CD4 and CD19 are present on distinct cells as expected, and within the CD19^+^ cells we can distinguish IgD-positive and -negative populations (Supplementary Fig. S7A).

The UMAP visualizations revealed noticeable differences in peripheral immune profiles between patient donors (Fig. 5E–H). Although the donors are under surveillance for breast cancer risk in the B-PREP clinic, they also potentially harbor other malignancies, which were unknown to us during the sample preparation, acquisition, and analysis stages. In the lymphocyte gate of both PBMC panels, a distinct cluster of B lymphocytes was apparent for patient 3146 but absent for the other two donors (Fig. 5E–G). After unblinding, we discovered that this patient had a chronic lymphoblastic leukemia diagnosis. Likewise, a population of CD4^+^ T cells was abundant in the PBMC sample from patient 3156 and absent from the other two donor samples (Fig. 5E); we subsequently learned that patient 3156 had been diagnosed with T-cell lymphoma.

### Batch Correction and Harmony Integration

**FIGURE 6:**
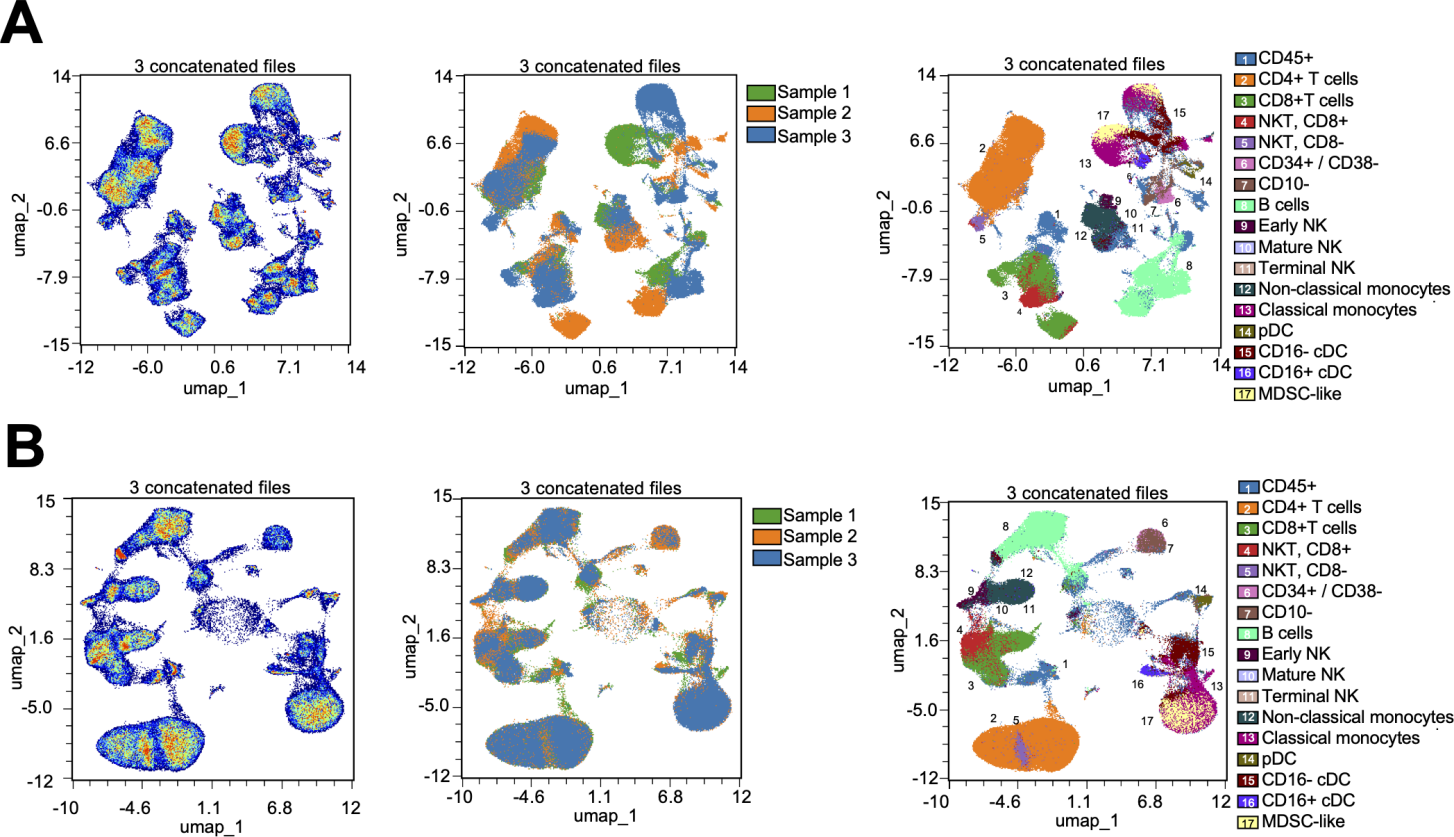
BMC panel harmony integration for sample-specific batch correction. **A,** UMAP coordinates generated from concatenated files of 50,000 CD45^+^, live, singlets from each of 3 BMC donors demonstrate clustering of cells in two-dimensional space (left); however, these data show a strong sample-specific batch effect (middle) and poor cell type-specific clustering (right). **B,** Following integration using Harmony, new UMAP coordinates are generated (left), the data from 3 donors colocalize (middle), and cells annotated as the same cell type cluster in two-dimensional space (right).

Finally, we sought to gain a deeper understanding of the data generated from three BMC samples. Initial analysis suggested that UMAP positioning was driven by cell types (as expected) as well as by donor sample, which was unexpected (Fig. 6A). We attributed this apparent donor-specific separation to technical variation because analysis of each sample was separated by more than seven weeks (see Materials and Methods). To correct for this, we adapted an algorithm (Harmony) for batch correction that was originally developed for the analysis of single-cell RNA-sequencing data (20, 29). Specifically, we wrote an R pipeline that performs Harmony batch correction within the Seurat framework. Harmony implements a soft *k*-means clustering algorithm to project cells into a shared embedding with the goal of grouping cells by cell type rather than by sample-specific conditions. After performing Harmony integration, the resulting UMAP improved the positioning of datapoints by cell type while decreasing the separation between donors (Fig. 6B).

We justified the implementation of Harmony to the BMC sample analysis by comparing the LISI for the cells in each UMAP (Figs. 5A–D and 6A). LISI is an objective metric of integration that calculates the number of “batches” that exist within the neighborhood of each cell in UMAP space (20). The LISI will range from 1 to the total number of batches (i.e., samples) in the analysis. In this study, a minimum LISI of 1 (no integration) and a maximum LISI of 3 (complete integration) could be achieved for each UMAP because we analyzed three samples.

As a measure of data integration, we assessed the proportions of cells with a high LISI score (>2, see Materials and Methods). As shown in Supplementary Fig. S8, a low proportion of cells from the BMC samples had a high LISI score (average 15.0% for the three BMC samples shown in Fig. 6A). These low LISI scores indicate that cells clustered by sample in the UMAP space, suggesting a batch effect in the bone marrow data. In contrast, Supplementary Fig. S8 shows higher LISI scores of the PBMC samples, indicating good integration (average 34.8% across the four PBMC UMAPs shown in Fig. 5A–D). Notably, LISI for Donor 3146 was 2.57-fold lower compared with the other two PBMC donors in the UMAPs that included lymphocytes (Fig. 5A–C), but only 1.32-fold lower in the UMAP without lymphocytes (Fig. 5D). We ascribed the reduced proportion of high LISI cells to the fact that Donor 3146 had a B-cell lymphoma, represented as a cluster unique to that donor in the UMAP (Fig 5E). In the UMAP without lymphocytes, >40% of cells had high LISI scores for all three samples; furthermore, removing Donor 3146 from the UMAP also increased the average proportion of high LISI cells (Supplementary Fig. S8). Together, these results support that the BMC data suffered from batch effects resulting in clustering by sample in UMAP space, whereas the PBMC data were well integrated with the exception of a biologically important B-cell lymphoma–specific cluster. These findings justify our implementation of Harmony specifically for the BMC data and demonstrate that algorithms developed for high-dimensional single-cell sequencing data can be successfully applied to high-dimensional flow cytometry data to improve the biological interpretability.

### Discussion

In this study, we developed a resource for high-dimensional flow cytometry–based analysis of human hematopoietic and peripheral immune cells as well as markers of activation, differentiation, and exhaustion at high throughput and low cost in a time-efficient manner. By incorporating a large number of markers into each panel and improving data visualization and batch correction strategies, we enable unbiased analysis and the potential identification of novel immune phenotypes with translational and clinical relevance. While various flow cytometric panels have been developed (6–11, 17, 18), our innovation lies in the incorporation of a large number of markers for deep immunoprofiling of small sample volumes, coupled with refined data visualization and batch correction analysis strategies. A key advantage of our approach is the simplicity and efficiency of the staining protocols, as these panels exclusively rely on cell-surface proteins.

The utilization of spectral flow cytometry, characterized by a substantial number of markers in each of these panels, also presents distinct considerations compared with conventional flow cytometry. For example, while recognizing the value of full-minus-one (FMO) controls in certain scenarios, the challenges arising from limited clinical sample availability and the inclusion of numerous markers in a single staining panel should be emphasized. In our study, the feasibility of running FMOs for all markers was hindered by these constraints. Consequently, the importance of single-color and unlabeled controls becomes paramount in experimental design when faced with limited sample availability. We encourage users to exercise their discretion in deciding whether to include FMOs for all or selected markers in their experimental design.

Concordance in cell populations and markers between panels indicates the reliability of our panel design and serves as an internal control for sample consistency. This consistency also allows for comparisons between panels, aiding in the interpretation of results across different experimental conditions or timepoints. In our data analysis, we employed heat map visualization and dimensionality reduction using UMAPs to visualize distinct clusters within the data. This visualization allowed for the identification of differences between samples and facilitated the subsequent examination of specific markers. Furthermore, we show how batch correction algorithms developed for single-cell sequencing can be successfully applied to spectral flow cytometry data to generate visualizations that better capture biological (rather than technical) features. Specifically, we found that the LISI scoring algorithm provides a useful metric to quantify sample integration. Cells with a high LISI score are well integrated, so if the proportion of cells with a high LISI score is small (e.g., <20%), then Harmony integration may be warranted. However, a notable limitation of the Harmony algorithm is its capacity to overintegrate data, thereby masking true biological differences. For example, a biologically meaningful B-cell lymphoma–specific cluster may be overcorrected by running Harmony making it difficult to discern from a healthy sample. It is ultimately up to user discretion to determine the optimal implementation of tools to derive biological insights. We suggest that users implement LISI after initial UMAP coordinate generation and determine the proportion of well-integrated cells (with a high LISI score) to determine whether Harmony integration adds value within their experimental context. These strategies facilitate biologically meaningful representation and interpretation of the data while generating a comprehensive understanding of the underlying cellular heterogeneity.

Our panels were developed for use with cryopreserved PBMCs and bone marrow samples. The use of frozen samples aligns with common practice in clinical settings, where the ability to collect and store PBMCs and BMCs offers practical advantages for large-scale and longitudinal studies involving patient samples. Nevertheless, we recognize that this practice leads to loss of some cell types including granulocytes and neutrophils, which is a limitation of this method. We envision that these panels could be adapted for studies of fresh whole blood and bone marrow samples to provide important information about these other cell types. Such a protocol would require proper panel design and optimization.

As with most studies involving analysis of donor specimens, we recognize inherent variability across donor populations. Hence, an advantage of our pipeline is that we intentionally incorporate some marker overlap between PBMC panels, and employ several data visualization and analysis strategies to account for biological and technical variability. For example, we demonstrate reproducible panel performance through concordance of marker expression and cell populations between distinct panels. We also provide new computational approaches to resolve batch-to-batch variability. Although the panels were validated using a limited number of donor samples, our approaches are designed so that users can resolve variability across large donor cohorts. At this time, our method is not intended to be used as a diagnostic tool but rather, to provide a deep understanding of hematopoietic and peripheral immune landscapes at the single-cell level and as such, should provide a powerful tool for research and biological discovery.

## Supplementary Material

Table S1 Table S1. T/B Panel Reagents. Information and concentration of indicated fluorophore-conjugated antibodies used to label PBMCs in these studies.

Table S2 Table S2. M/N/D Panel Reagents. Information and concentration of indicated fluorophore-conjugated antibodies used to label PBMCs in these studies.

Table S3 Table S3. BMC Panel Reagents. Information and concentration of indicated fluorophore-conjugated antibodies used to label BMCs in these studies.

Table S4 Table S4. PBMC and BMC Sample Viability. Viability of PBMC and BMC donor samples calculated upon thawing, represented as a percentage of Fixable-ViaDyeRed negative cells. Samples were gated for all events, singlets, and non-RBCs.

Figure S1 Figure S1. PBMC Antibody Staining Performance. (A, B) Histograms showing fluorescence intensity and cell counts when antibodies were used as single-color controls (SC; blue lines) overlaid with staining performance within the full antibody cocktail (multicolor, MC; black lines), in the T/B panel (A) and M/N/D panel (B). The same PBMC donor sample was used for (A and B) and data represent cells gated as singlets, non-RBC, live cells, and the appropriate lymphocyte or monocyte scatter gates. Data were normalized using the time gate to select equal numbers of cells for SC and MC for each marker.

Figure S2 Figure S2. Unmixing Accuracy Assessment. NxN permutations showing density plots of the same marker on the x-axis and every other fluorochrome plotted on the y-axis. Plots were manually examined for accuracy of unmixing. (A) T/B panel; (B) M/N/D panel; (E, F) BMC panel. The same PBMC donor sample was used for (A and B) and data represent cells gated as singlets, non-RBC, and live cells. (C) Density plot of CD159c-BYG575 vs CD19-BYG710 after automated unmixing. (D) Density plot of CD159c-BYG575 vs CD19-BYG710 after applying additional manual compensation of -2.79 using the SpectroFlo compensation tool. (F) NxN plots after removing antibody aggregates in NovaFluor Blue 610-70S – CD19. (G) Antibody aggregates are manually removed through the use of a NOT gate in the aberrant population.

Figure S3 Figure S3. T/B and M/N/D Staining and Panel Consistency in Cell Type Detection. (A) Histograms showing expression of the 3 antibodies used in both the T/B and M/N/D PBMC panels (B) Bar graphs showing average stain index calculated using the median and standard deviation of the positive and negative peaks for each of the 3 antibodies used in both the T/B and M/N/D PBMC panels (n=3 donor samples). Stain Index= ((〖Median〗_positive-〖Median〗_negative))/〖2*SD〗_negative . Error bars represent SEM and statistical significance was calculated by t-test with p>0.05 being n.s. (not significant). (C) Ratio of lymphocytes (upper portion of graph, separated by the dotted line) and NK cells (lower portion of graph) to total CD45+ cells for each indicated donor in the T/B panel (green) and M/N/D panel (blue). Data were analyzed by two-way ANOVA with no significant differences calculated due to panel or cell type assessed.

Figure S4 Figure S4. T Cell Activation and Exhaustion. Density plots of T cell activation and exhaustion markers on CD3+ PBMCs concatenated from three donor samples.

Figure S5 Figure S5. NK cell Marker Expression. Density plots of NK cell functional marker expression by subtype. Columns depict NK cell subtype (all NK, Mature NK, Early NK, and Terminal NK cells), and rows show individual markers. The first row shows the gating strategy used to identify each NK cell subtype from the total NK cells parent population. Gates indicate positive populations, with the percentage of the parental population displayed in each panel.

Figure S6 Figure S6. Detection of Rare and Unexpected Cell Populations. Density plots of CD4+/CD8+ double-positive T cells (A), Basophils (B) and ILCs (C) identified in the PBMC samples. Gates indicate positive populations, with the percentage of the parental population displayed in each panel. Both concatenated and individual donor plots are shown.

Figure S7 Figure S7. UMAP Marker Positioning. (A-E) UMAPs showing marker expression patterns in PBMC and BMC panels. Marker expression intensity is indicated by the scale bar to the right of each plot, where red is high, and blue is low. Data are derived from concatenated events from all 3 donors in the PBMC panels (A-D), and concatenated events from all 3 donors in the BMC panel (E). Insert in bottom right of each figure shows canonically gated major cell populations. Populations 10, 11, and 12 share CD16 expression, and as such occupy similar locations in UMAP space.

Figure S8 Figure S8. Pre-integration LISI scores for PBMC and BMC UMAPs. Dot plot indicating proportion of cells with a high Local Inverse Simpson Index (LISI) score in concatenated UMAPs. To calculate this proportion, we set a threshold of LISI > 2 in those cases where scores could range from 1-3 (“BMC” and “PBMC (All Donors)”; UMAPs shown in Fig. 6A and 5A-D), or LISI > 1.667 in those cases where scores could range from 1-2 (“PBMC (Excluding Donor 3146)”, UMAPs not shown). Circular data points represent the proportion of LISI-high cells per indicated donor within each UMAP; red squares indicate the average proportion of LISI-high cells among all donor cells in the respective UMAP. For PBMC UMAPs, “All Donors” represents the inclusion of all three PBMC samples, while “Excluding Donor 3146” excludes a donor who had a B cell lymphoma, demonstrating how biological differences can impact integration.
