# Supplementary material for: Spectral Flow Cytometry Methods and Pipelines for Comprehensive Immunoprofiling of Human Peripheral Blood and Bone Marrow
Source: Cancer Res Commun. 2024 Mar 25;4(3):895–910. doi: 10.1158/2767-9764.CRC-23-0357 (PMC10962315; doi:10.1158/2767-9764.CRC-23-0357)
Supplement: Table S1 — T/B Panel Reagents. Information and concentration of indicated fluorophore-conjugated antibodies used to label PBMCs in these studies. [file crc-23-0357-s01.pdf]

Table S1: T/B Panel Reagents

Marker	Fluorophore	Clone	Source	Identifier	ng/1M cells	Purpose
**CD45**	cFluor B548	2D1	Cytek Biosciences	Cat# RC-00025	125	Leukocytes
**CD279 (PD-1)**	BB515	EH12.1	BD Biosciences	Cat# 564494, RRID:AB_2738827	125	T cell inhibitory receptor
**CD196 (CCR6)**	BB700	11A9	BD Biosciences	Cat# 566477, RRID:AB_2744303	125	Chemokine receptor; T & B cell differentiation
**CD185 (CXCR5)**	PerCP-eFluor 710	MU5UBEE	ThermoFisher	Cat# TF 46-9185-42, RRID:AB_2573877	30	Chemokine receptor; T cell differentiation
**CD25**	cFluor BYG575	BC96	Cytek Biosciences	Cat# RC-00044	100	Regulatory T cells
**CD4**	cFluor YG584	SK3	Cytek Biosciences	Cat# R7-20041, RRID:AB_2885083	50	CD4 T and NKT-Like cells
**CD152 (CTLA-4)**	cFluor BYG610	BN13	Cytek Biosciences	Cat# RC-00045	250	T cell inhibitory receptor
**CD19**	PE Fire 640	HIB19	BioLegend	Cat# 302274, RRID:AB_2860773	31.25	B cells
**CD366 (TIM-3)**	cFluor BYG667	F38-2E2	Cytek Biosciences	Cat# RC-00046	250	T cell and NK Cell exhaustion marker
**TCRγδ**	cFluor BYG710	B1	Cytek Biosciences	Cat# RC-00047	300	Pan γδ T cell
**CD197 (CCR7)**	cFluor BYG781	G043H7	Cytek Biosciences	Cat# RC-00048	200	T cell differentiation
**CD183 (CXCR3)**	cFluor R659	G025H7	Cytek Biosciences	Cat# RC-00049	200	Chemokine receptor; DC, T & B cell differentiation
**CD45RA**	cFluor R685	HI100	Cytek Biosciences	Cat# RC-00050	125	T cell & DC differentiation
**CD38**	cFluor R720	LS198-4-3	Cytek Biosciences	Cat# R7-20061	125	Monocyte, DC, T & B cell activation/differentiation
**HLA-DR**	cFluor R780	L243	Cytek Biosciences	Cat# RC-00051	20	T cell & monocyte activation, NK & DC lineage
**CD3**	cFluor R840	SK7	Cytek Biosciences	Cat# RC-00052	50	Pan T cell, NKT-Like cells
**CD194 (CCR4)**	BV421	L291H4	BioLegend	Cat# 359414, RRID:AB_2562435	62.5	TH2 marker
**IgD**	cFluor V450	IA6-2	Cytek Biosciences	Cat# RC-00053	125	B cell differentiation
**CD223 (LAG-3)**	BV480	T47-5330	BD Biosciences	Cat# 746609, RRID:AB_2743890	500	T cell & NK exhaustion
**TCRαβ**	BV510	IP26	BioLegend	Cat# 306734, RRID:AB_2650821	125	Pan αβ T cell
**CD20**	BV570	2H7	BioLegend	Cat# 302332, RRID:AB_2563805	125	B cells
**CD161**	cFluor V610	HP-3G10	Cytek Biosciences	Cat# RC-00054	125	TH17, NKT-Like cells
**CD27**	BV650	O323	BioLegend	Cat# 302828, RRID:AB_2562096	62.5	T & B cell differentiation
**CD127 (IL-7Ra)**	BV711	A019D5	BioLegend	Cat# 351328, RRID:AB_2562908	62.5	Cytokine receptor; T cell differentiation
**CD28**	BV750	CD28.2	BioLegend	Cat# 302970, RRID:AB_2876593	93.75	T cell & NK differentiation
**CD8**	BV785	SK1	BioLegend	Cat# 344740, RRID:AB_2566202	31.25	CD8 T, NK, & NKT-Like cells
**Fixable**	ViaDyeRed	Viability	Cytek Biosciences	Cat# R7-60008		Viability

Table S1. T/B Panel Reagents. Information and concentration of indicated fluorophore-conjugated antibodies used to label PBMCs in these studies.
