# Supplementary material for: Spectral Flow Cytometry Methods and Pipelines for Comprehensive Immunoprofiling of Human Peripheral Blood and Bone Marrow
Source: Cancer Res Commun. 2024 Mar 25;4(3):895–910. doi: 10.1158/2767-9764.CRC-23-0357 (PMC10962315; doi:10.1158/2767-9764.CRC-23-0357)
Supplement: Table S2 — M/N/D Panel Reagents. Information and concentration of indicated fluorophore-conjugated antibodies used to label PBMCs in these studies. [file crc-23-0357-s02.pdf]

## Table S2: M/N/D Panel Reagents

| Marker | Fluorophore | Clone | Source | Identifier | ng/1M cells | Purpose |
|---|---|---|---|---|---|---|
| **CD45** | cFluor B548 | 2D1 | Cytek Biosciences | Cat# RC-00025 | 125 | Leukocytes |
| **CD11b** | BB515 | ICRF44 | BD Biosciences | Cat# 564517, RRID:AB_2744271 | 62.5 | Myeloid |
| **CD20** | cFluor B675 | 2H7 | Cytek Biosciences | Cat# RC-00030 | 125 | B cells |
| **CD366 (TIM-3)** | BB700 | 344823 | BD Biosciences | Cat# 747957, RRID:AB_2872418 | 125 | T cell & NK cell exhaustion |
| **CD159c (NKG2C)** | cFluor BYG575 | S19005E | Cytek Biosciences | Cat# RC-00040 | 3.125 | NK cell differentiation |
| **CD337 (NKp30)** | cFluor BYG610 | P30-15 | Cytek Biosciences | Cat# RC-00056 | 250 | NK cell differentiation |
| **CD33** | cFluor BYG667 | WM53 | Cytek Biosciences | Cat# RC-00041 | 0.5 | Myeloid differentiation |
| **CD19** | cFluor BYG710 | HIB19 | Cytek Biosciences | Cat# R7-20009 | 15 | B cells |
| **CD94 (KLRD1)** | cFluor BYG781 | DX22 | Cytek Biosciences | Cat# RC-00043 | 50 | NK cell differentiation |
| **CD335 (NKp46)** | cFluor R659 | 9.00E+02 | Cytek Biosciences | Cat# RC-00055 | 100 | NK cell activation |
| **CD314 (NKG2D)** | cFluor R685 | 1D11 | Cytek Biosciences | Cat# RC-00028 | 125 | NK cell differentiation |
| **CD14** | cFluor R720 | RMO52 | Cytek Biosciences | Cat# RC-00029 | 250 | Monocyte, Myeloid differentiation |
| **HLA-DR** | cFluor R780 | L243 | Cytek Biosciences | Cat# RC-00051 | 20 | T cell & monocyte activation, NK & DC lineage |
| **CD3** | cFluor R840 | SK7 | Cytek Biosciences | Cat# RC-00052 | 50 | Pan T cell, NKT-Like cells |
| **CD336 (NKp44)** | BV421 | p44-8 | BD Biosciences | Cat# 744299, RRID:AB_2742129 | 250 | NK cell activation |
| **CD123** | cFluor V450 | 6H6 | Cytek Biosciences | Cat# RC-00026 | 125 | Plasmacytoid dendritic cells, basophils |
| **CD7** | BV480 | M-T701 | BD Biosciences | Cat# 566119, RRID:AB_2739521 | 31.25 | NK cells |
| **CD5** | BV510 | L17F12 | BioLegend | Cat# 364018, RRID:AB_2565728 | 15.625 | Dendritic cell differentiation |
| **CD16** | BV570 | 3G8 | BioLegend | Cat# 302036, RRID:AB_2632790 | 50 | Monocyte, NK cell, & DC differentiation |
| **CD163** | cFluor V610 | GHI/61 | Cytek Biosciences | Cat# RC-00027 | 125 | Monocyte differentiation |
| **CD1c** | BV650 | L161 | BioLegend | Cat# 331542, RRID:AB_2800866 | 31.25 | Dendritic cells, NKT-Like cells |
| **CD158 (KIR2DL1/S1/S3/S5)** | BV711 | HP-MA4 | BD Biosciences | Cat# 752507, RRID:AB_2917499 | 125 | NK cell activation |
| **CD141** | BV750 | 1A4 | BD Biosciences | Cat# 747244, RRID:AB_2871963 | 250 | Dendritic cell differentiation |
| **CD56** | BV785 | 5.1H11 | BioLegend | Cat# 362550, RRID:AB_2566059 | 62.5 | Pan NK cell, γδ T cell activation |
| **Fixable** | ViaDyeRed | Viability | Cytek Biosciences | Cat# R7-60008 | | Viability |

**Table S2. M/N/D Panel Reagents**. Information and concentration of indicated fluorophore-conjugated antibodies used to label PBMCs in these studies.
