# Supplementary material for: Spectral Flow Cytometry Methods and Pipelines for Comprehensive Immunoprofiling of Human Peripheral Blood and Bone Marrow
Source: Cancer Res Commun. 2024 Mar 25;4(3):895–910. doi: 10.1158/2767-9764.CRC-23-0357 (PMC10962315; doi:10.1158/2767-9764.CRC-23-0357)
Supplement: Table S3 — BMC Panel Reagents. Information and concentration of indicated fluorophore-conjugated antibodies used to label BMCs in these studies. [file crc-23-0357-s03.pdf]

Table S3: BMC Panel Reagents

Marker	Fluorophore	Clone	Source	Identifier	ng/1M cells	Purpose
**CD74**	BV421	LN2	BD Biosciences	Cat# 743731, RRID:AB_2741705	500	Antigen presentation
**CD123 (IL3RA)**	cFluor V450	6H6	Cytek Biosciences	Custom	100	Plasmacytoid dendritic cells
**CD11c**	BV480	B-Ly6	BD Biosciences	Cat# 566135, RRID:AB_2739534	250	Dendritic cells
**TCRαβ**	BV510	IP26	BioLegend	Cat# 306734, RRID:AB_2650821	500	Pan αβ T cell
**CD14**	cFluor V547	63D3 or M5E2	Cytek Biosciences	Custom	250	Monocytes and MDSC-like
**CD8**	BV570	RPA-T8	BioLegend	Cat# 301038, RRID:AB_2563213	50	CD8 T, NK, and NKT-Like cells
**CD80**	BV605	2D10	BioLegend	Cat# 305225, RRID:AB_11123909	750	Modulation of immune response
**CD86**	BV650	IT2.2	BioLegend	Cat# 305428, RRID:AB_2563823	250	Modulation of immune response
**CD206 (MRC1)**	BV711	15-2	BioLegend	Cat# 321136, RRID:AB_2687200	500	Monocyte differentiation
**CD127**	BV750	A019D5	BioLegend	Cat# 351369, RRID:AB_2892427	100	Cytokine receptor; T cell differentiation, innate lymphoid cells
**CD274 (PD-L1)**	BV785	29E.2A3	BioLegend	Cat# 329736, RRID:AB_2629582	125	Immunosuppressive monocytes
**CD11b**	BB515	ICRF44	BD Biosciences	Cat# 564517, RRID:AB_2744271	25	Myeloid differentiation and MDSC-like
**CD45**	cFluor B548	HI30	Cytek Biosciences	R7-20292	50	Leukocytes
**CD19**	NovaFluor Blue 610 70S	HIB19	ThermoFisher	Cat# H004T03B06, RRID:AB_2896495	500	B cells
**CD16**	cFluor B677	3G8	Cytek Biosciences	Custom	100	Non-classical monocyte, NK and DC differentiation
**CD34**	BB700	8G12	BD Biosciences	Cat# 746104, RRID:AB_2743475	125	Hematopoietic stem and progenitor cells
**CD366 (TIM-3)**	PerCP-eFluor 710	F38-2E2	ThermoFisher	Cat# 46-3109-42, RRID:AB_11041821	30	T cell and NK cell exhaustion marker
**CD20**	BB755	2H7	BD Biosciences	Custom	250	B cells
**CD25**	cFluor BYG575	BC96	Cytek Biosciences	Custom	150	Regulatory T cells
**CD4**	cFluor YG584	SK3	Cytek Biosciences	Cat# R7-20041, RRID:AB_2885083	10	CD4 T and NKT-Like cells
**CD90**	cFluor BYG610	5E10	Cytek Biosciences	Custom	100	Hematopoietic stem cells
**CD56**	cFluor YG610	BA-19	Cytek Biosciences	Custom	500	Pan NK cell, γδ T cell activation
**CD3**	PE/Fire 640	SK7	BioLegend	Cat# 344860, RRID:AB_2860897	50	Pan T cell, NKT-Like cells
**CD135 (FLT3)**	PE/Cyanine5	BY10A4H2	BioLegend	Cat# 313310, RRID:AB_2278486	250	Myeloid vs. erythroid progenitors
**TCRγ𝛿**	cFluor BYG710	B1	Cytek Biosciences	Custom	250	Pan γδ T cell
**CD163**	cFluor BYG750	GHI/61	Cytek Biosciences	Custom	200	Monocyte differentiation
**CD197 (CCR7)**	cFluor BYG781	G043H7	Cytek Biosciences	Custom	250	T cell differentiation
**CD33**	cFluor R659	WM53	Cytek Biosciences	Custom	10	Myeloid differentiation and MDSC-like
**CD45RA**	cFluor R685	HI100	Cytek Biosciences	Custom	100	Multi-lymphoid progenitor; T cell & DC differentiation
**HLA-DR**	cFluor R720	L243	Cytek Biosciences	Custom	10	T cell & monocyte activation, NK & DC lineage, antigen presentation
**CD10**	cFluor R780	HI10a	Cytek Biosciences	Custom	400	B/NK precursors
**CD38**	cFluor R840	HIT-2	Cytek Biosciences	Custom	200	Progenitor, monocyte, DC, T cell, and B cell status
**Fixable**	ViaDye Red	Viability	Cytek Biosciences	Cat# R7-60008		Viability

Table S3. BMC Panel Reagents. Information and concentration of indicated fluorophore-conjugated antibodies used to label BMCs in these studies.
