# Supplementary material for: Spectral Flow Cytometry Methods and Pipelines for Comprehensive Immunoprofiling of Human Peripheral Blood and Bone Marrow
Source: Cancer Res Commun. 2024 Mar 25;4(3):895–910. doi: 10.1158/2767-9764.CRC-23-0357 (PMC10962315; doi:10.1158/2767-9764.CRC-23-0357)
Supplement: Table S4 — PBMC and BMC Sample Viability. Viability of PBMC and BMC donor samples calculated upon thawing, represented as a percentage of Fixable-ViaDyeRed negative cells. Samples were gated for all events, singlets, and non-RBCs. [file crc-23-0357-s04.pdf]

Table S4

PBMC – T/B Panel			PBMC – M/N/D Panel			BMC Panel	
Sample ID	Viability (%)		Sample ID	Viability (%)		Sample ID	Viability (%)
958	99.47		958	96.32		32822	88.87
3146	99.49		3146	99.19		20222	82.71
3156	98.8		3156	98.14		71422	97.62

Table S4. PBMC and BMC Sample Viability. Viability of PBMC and BMC donor samples calculated upon thawing, represented as a percentage of Fixable-ViaDyeRed negative cells. Samples were gated for all events, singlets, and non-RBCs.
