# Supplementary material for: Spectral Flow Cytometry Methods and Pipelines for Comprehensive Immunoprofiling of Human Peripheral Blood and Bone Marrow
Source: Cancer Res Commun. 2024 Mar 25;4(3):895–910. doi: 10.1158/2767-9764.CRC-23-0357 (PMC10962315; doi:10.1158/2767-9764.CRC-23-0357)
Supplement: Figure S1 — PBMC Antibody Staining Performance. (A, B) Histograms showing fluorescence intensity and cell counts when antibodies were used as single-color controls (SC; blue lines) overlaid with staining performance within the full antibody cocktail (multicolor, MC; black lines), in the T/B panel (A) and M/N/D panel (B). The same PBMC donor sample was used for (A and B) and data represent cells gated as singlets, non-RBC, live cells, and the appropriate lymphocyte or monocyte scatter gates. Data were normalized using the time gate to select equal numbers of cells for SC and MC for each marker. [file crc-23-0357-s05.pdf]

Figure S1

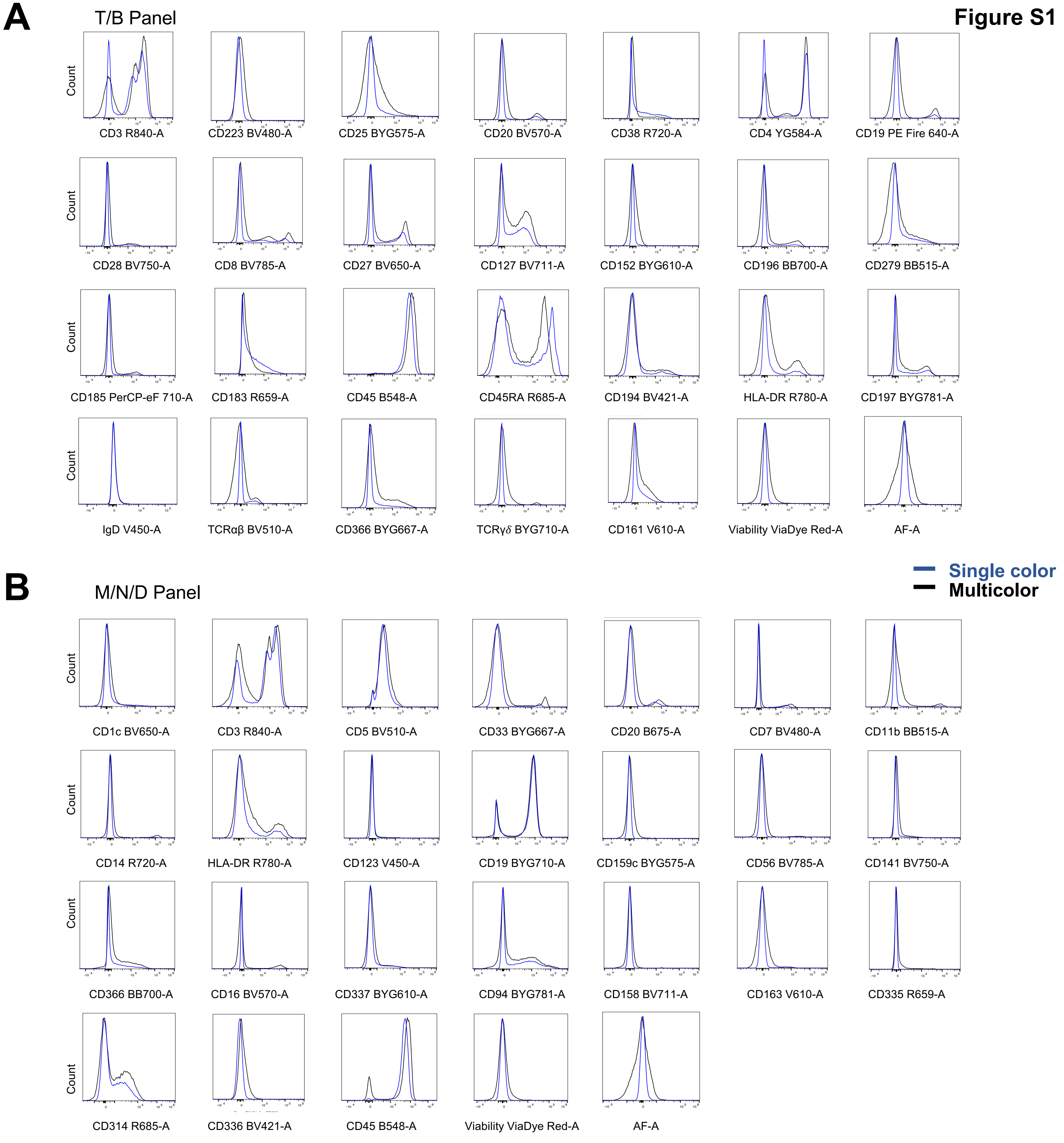

Figure S1. PBMC Antibody Staining Performance. (**A, B**) Histograms showing fluorescence intensity and cell counts when antibodies were used as single-color controls (SC; blue lines) overlaid with staining performance within the full antibody cocktail (multicolor, MC; black lines), in the T/B panel (**A**) and M/N/D panel (**B**). The same PBMC donor sample was used for (**A** and **B**) and data represent cells gated as singlets, non-RBC, live cells, and the appropriate lymphocyte or monocyte scatter gates. Data were normalized using the time gate to select equal numbers of cells for SC and MC for each marker.
