# Supplementary material for: Spectral Flow Cytometry Methods and Pipelines for Comprehensive Immunoprofiling of Human Peripheral Blood and Bone Marrow
Source: Cancer Res Commun. 2024 Mar 25;4(3):895–910. doi: 10.1158/2767-9764.CRC-23-0357 (PMC10962315; doi:10.1158/2767-9764.CRC-23-0357)
Supplement: Figure S2 — Unmixing Accuracy Assessment. NxN permutations showing density plots of the same marker on the x-axis and every other fluorochrome plotted on the y-axis. Plots were manually examined for accuracy of unmixing. (A) T/B panel; (B) M/N/D panel; (E, F) BMC panel. The same PBMC donor sample was used for (A and B) and data represent cells gated as singlets, non-RBC, and live cells. (C) Density plot of CD159c-BYG575 vs CD19-BYG710 after automated unmixing. (D) Density plot of CD159c-BYG575 vs CD19-BYG710 after applying additional manual compensation of -2.79 using the SpectroFlo compensation tool. (F) NxN plots after removing antibody aggregates in NovaFluor Blue 610-70S – CD19. (G) Antibody aggregates are manually removed through the use of a NOT gate in the aberrant population. [file crc-23-0357-s06.pdf]

Figure S2

A

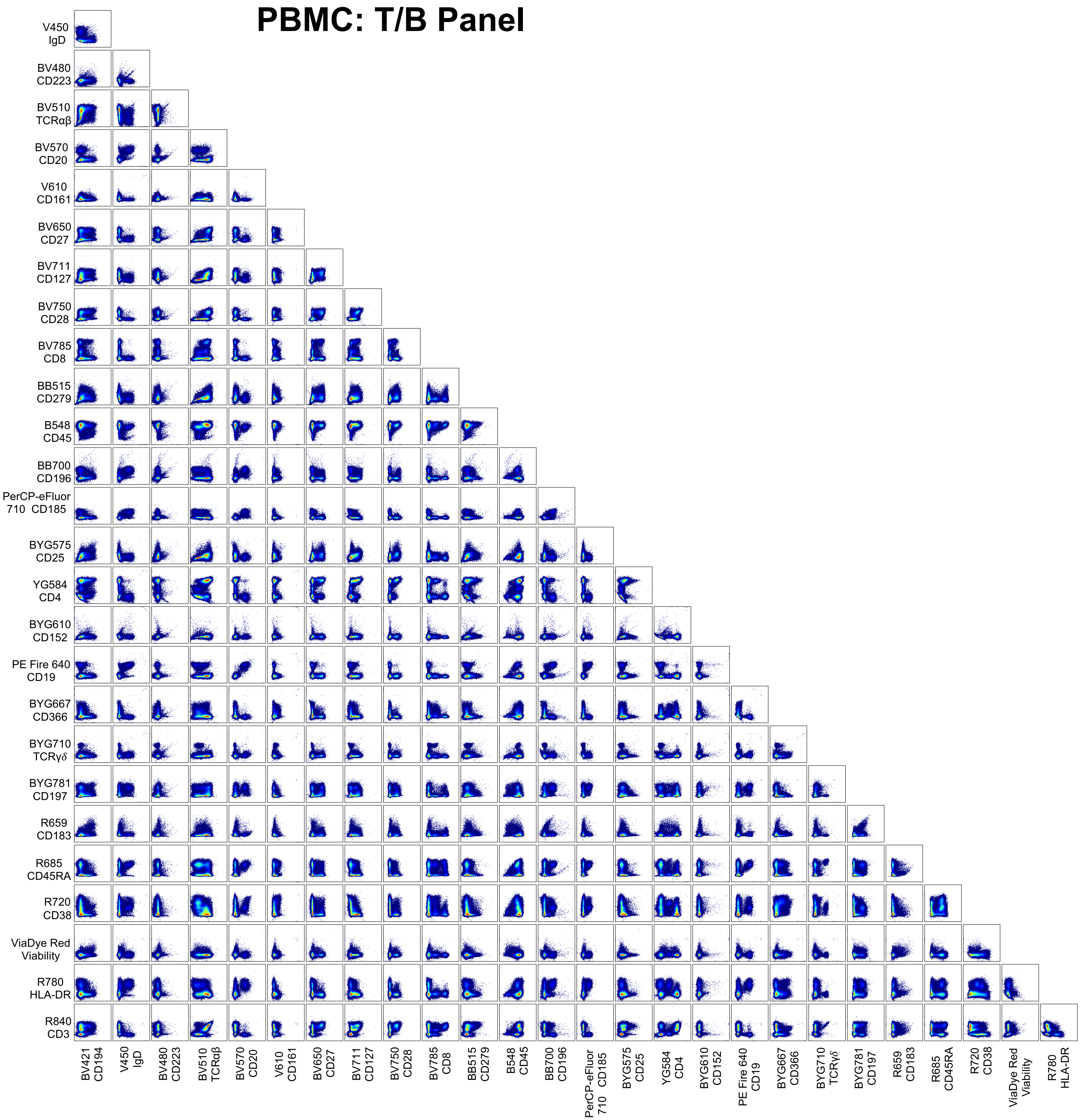
PBMC: T/B Panel
V450 IgD
BV480 CD223
BV510 TCRαβ
BV570 CD20
V610 CD161
BV650 CD27
BV711 CD127
BV750 CD28
BV785 CD8
BB515 CD279
B548 CD45
BB700 CD196
PerCP-eFluor 710 CD185
BYG575 CD25
YG584 CD4
BYG610 CD152
PE Fire 640 CD19
BYG667 CD366
BYG710 TCRγδ
BYG781 CD197
R659 CD183
R685 CD45RA
R720 CD38
ViaDye Red Viability
R780 HLA-DR
R840 CD3
BV421 CD194

B

PBMC: M/N/D Panel

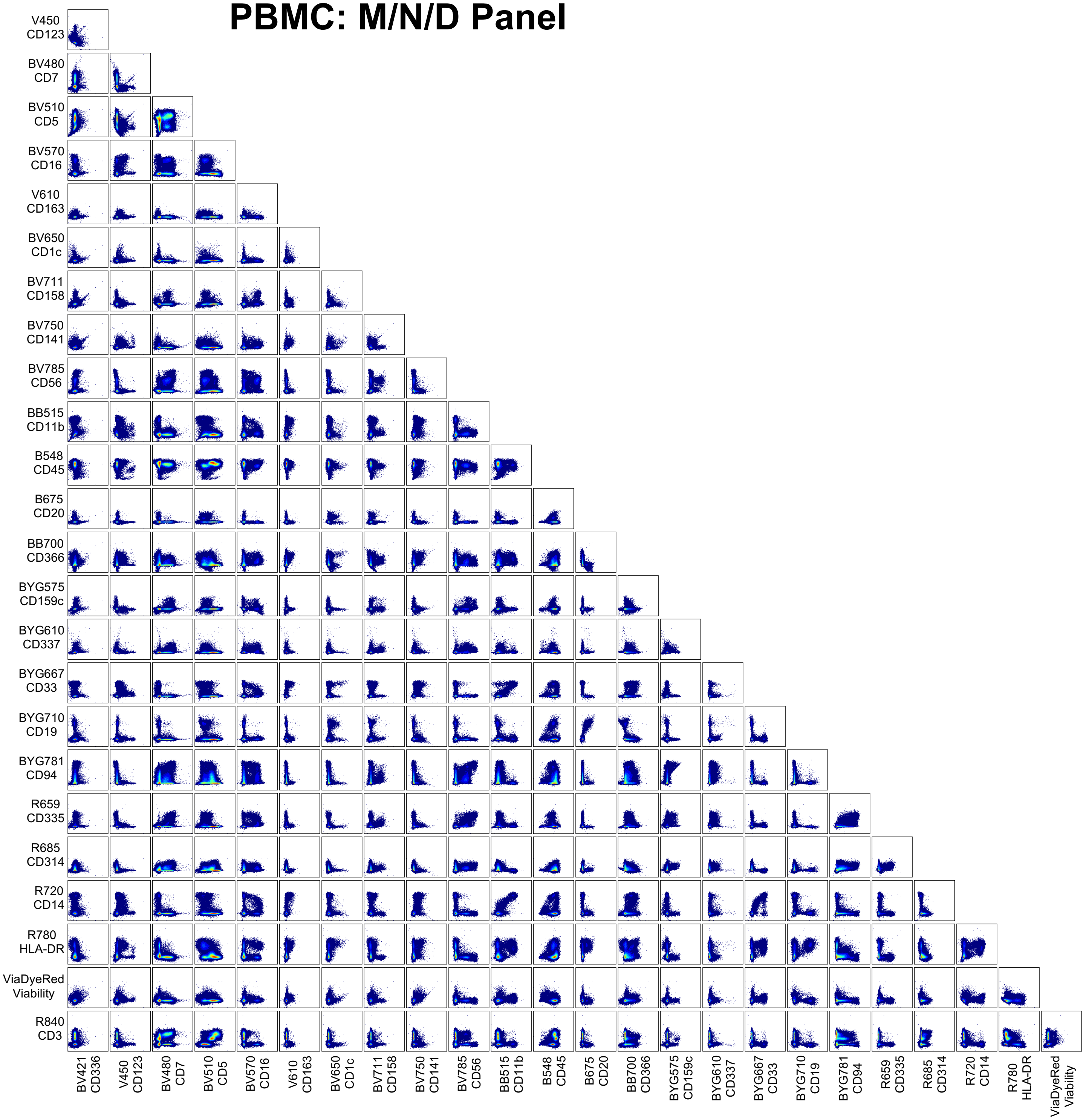

C

Original
(after unmixing)

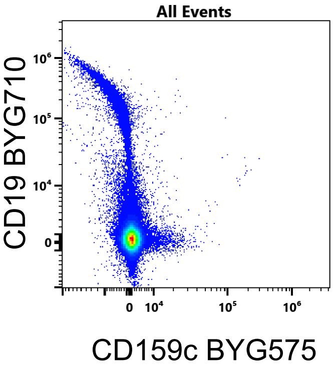

D

Additional compensation
(from BYG575 into BYG710: -2.79)

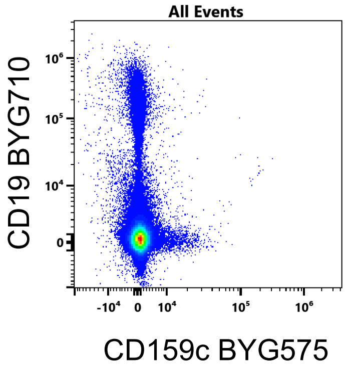

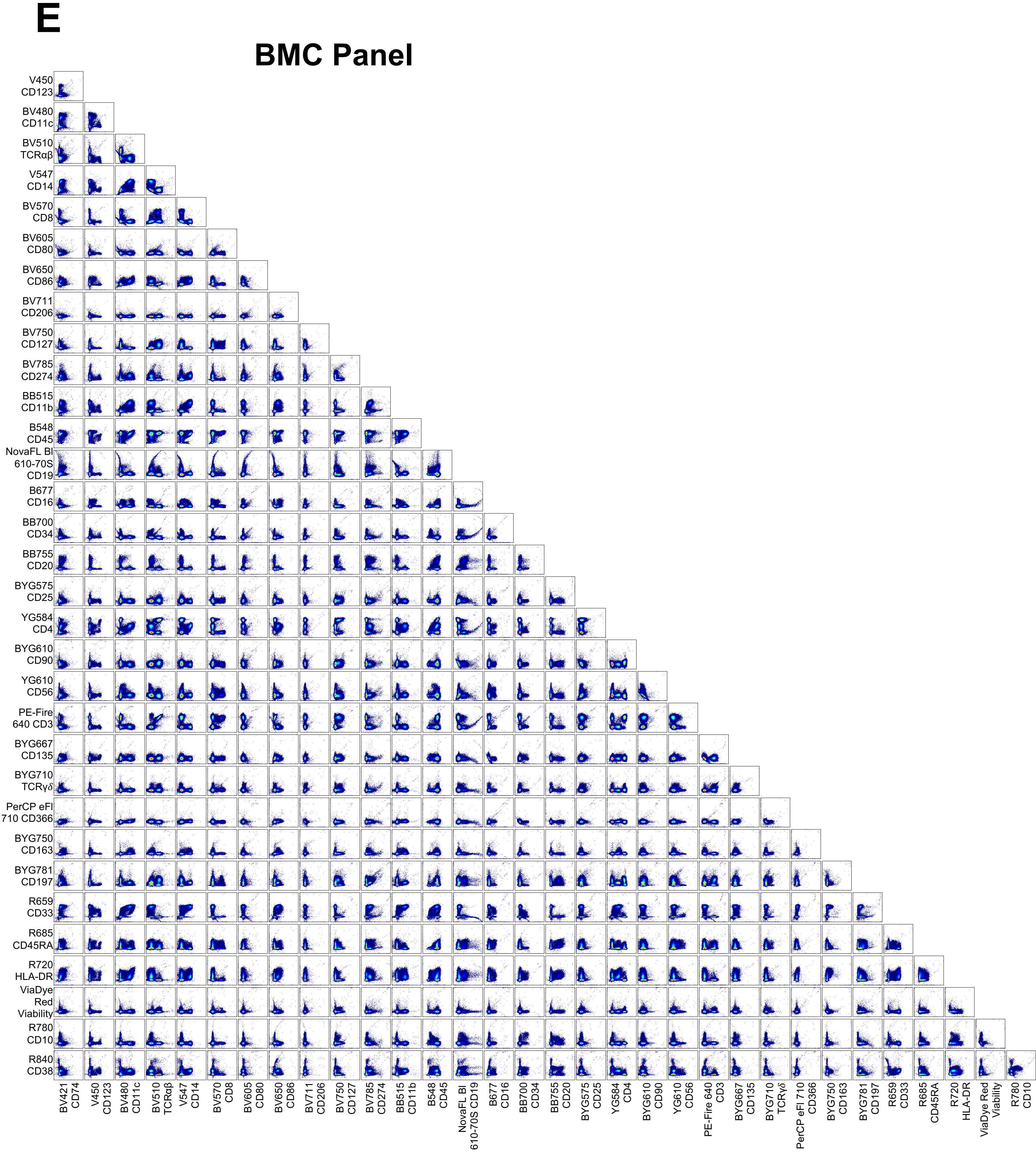
E
BMC Panel
V450 CD123
BV480 CD11c
BV510 TCRαβ
V547 CD14
BV570 CD8
BV605 CD80
BV650 CD86
BV711 CD206
BV750 CD127
BV785 CD274
BB515 CD11b
B548 CD45
NovaFL Bl 610-70S CD19
B677 CD16
BB700 CD34
BB755 CD20
BYG575 CD25
YG584 CD4
BYG610 CD90
YG610 CD56
PE-Fire 640 CD3
BYG667 CD135
BYG710 TCRγδ
PerCP eFl 710 CD366
BYG750 CD163
BYG781 CD197
R659 CD33
R685 CD45RA
R720 HLA-DR
ViaDye Red Viability
R780 CD10
R840 CD38
BV421 CD74

F

BMC Panel: Removal of aggregates

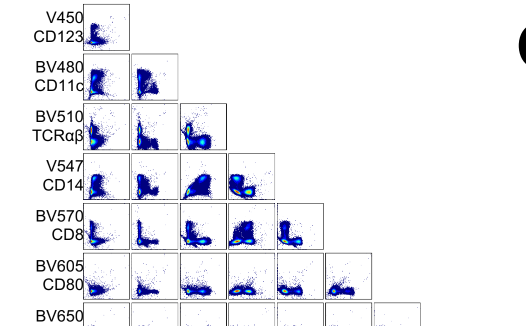
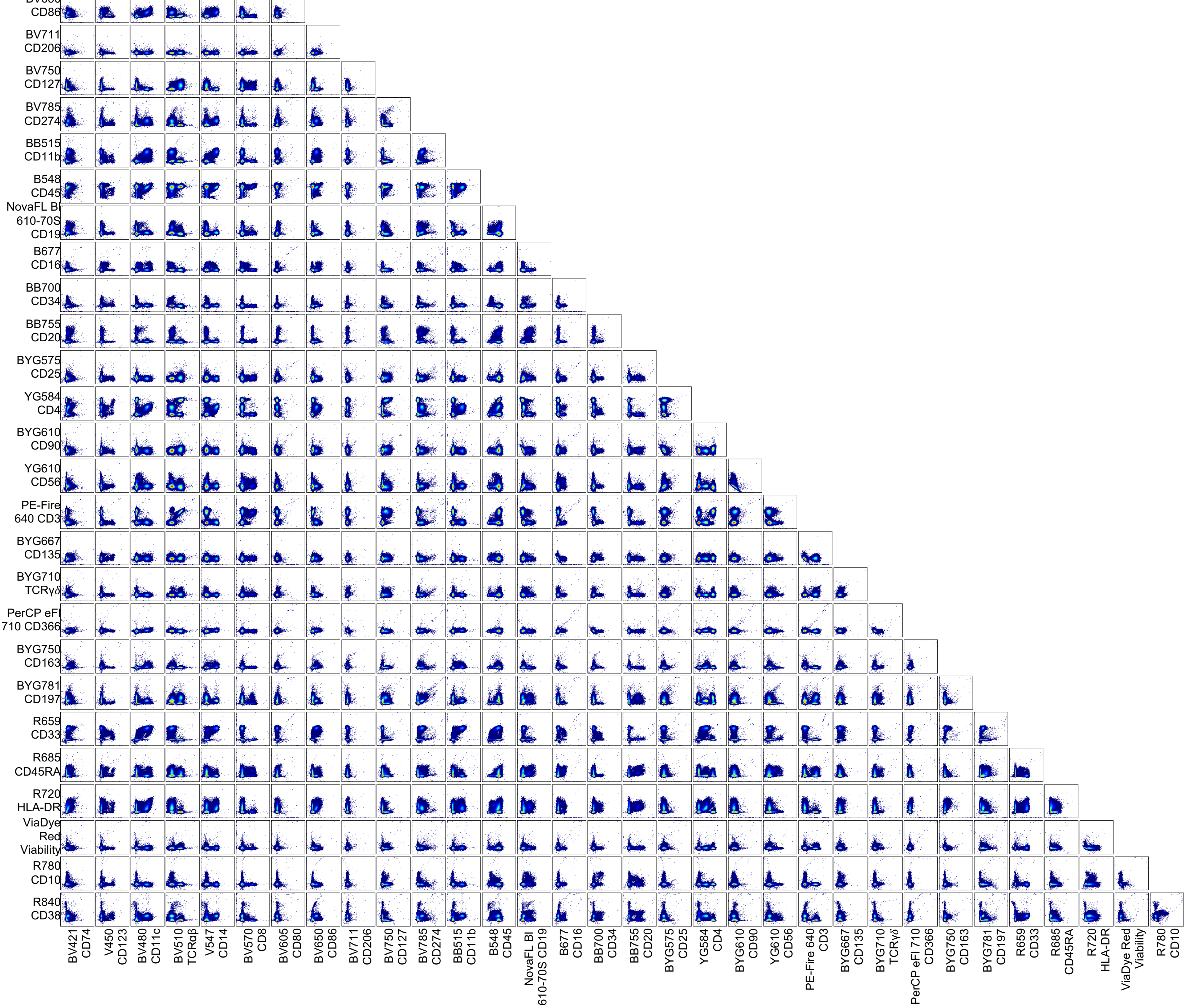

G

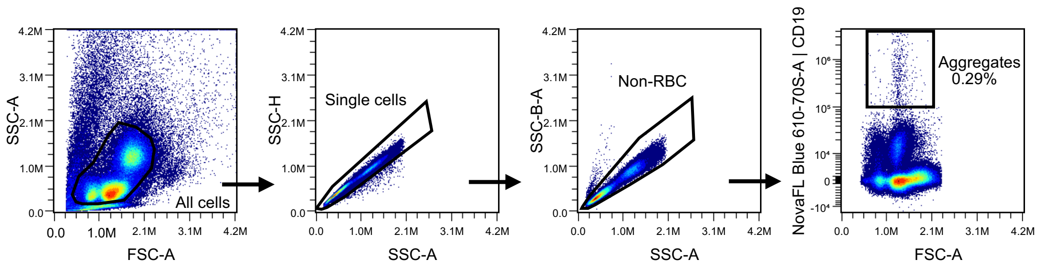

Figure S2. Unmixing Accuracy Assessment. NxN permutations showing density plots of the same marker on the x-axis and every other fluorochrome plotted on the y-axis. Plots were manually examined for accuracy of unmixing. (**A**) T/B panel; (**B**) M/N/D panel; (**E, F**) BMC panel. The same PBMC donor sample was used for (**A** and **B**) and data represent cells gated as singlets, non-RBC, and live cells. (**C**) Density plot of CD159c-BYG575 vs CD19-BYG710 after automated unmixing. (**D**) Density plot of CD159c-BYG575 vs CD19-BYG710 after applying additional manual compensation of -2.79 using the SpectroFlo compensation tool. (**F**) NxN plots after removing antibody aggregates in NovaFluor Blue 610-70S – CD19. (**G**) Antibody aggregates are manually removed through the use of a NOT gate in the aberrant population.
