# Supplementary material for: Spectral Flow Cytometry Methods and Pipelines for Comprehensive Immunoprofiling of Human Peripheral Blood and Bone Marrow
Source: Cancer Res Commun. 2024 Mar 25;4(3):895–910. doi: 10.1158/2767-9764.CRC-23-0357 (PMC10962315; doi:10.1158/2767-9764.CRC-23-0357)
Supplement: Figure S3 — T/B and M/N/D Staining and Panel Consistency in Cell Type Detection. (A) Histograms showing expression of the 3 antibodies used in both the T/B and M/N/D PBMC panels (B) Bar graphs showing average stain index calculated using the median and standard deviation of the positive and negative peaks for each of the 3 antibodies used in both the T/B and M/N/D PBMC panels (n=3 donor samples). Stain Index= ((〖Median〗_positive-〖Median〗_negative))/〖2*SD〗_negative . Error bars represent SEM and statistical significance was calculated by t-test with p>0.05 being n.s. (not significant). (C) Ratio of lymphocytes (upper portion of graph, separated by the dotted line) and NK cells (lower portion of graph) to total CD45+ cells for each indicated donor in the T/B panel (green) and M/N/D panel (blue). Data were analyzed by two-way ANOVA with no significant differences calculated due to panel or cell type assessed. [file crc-23-0357-s07.pdf]

Figure S3

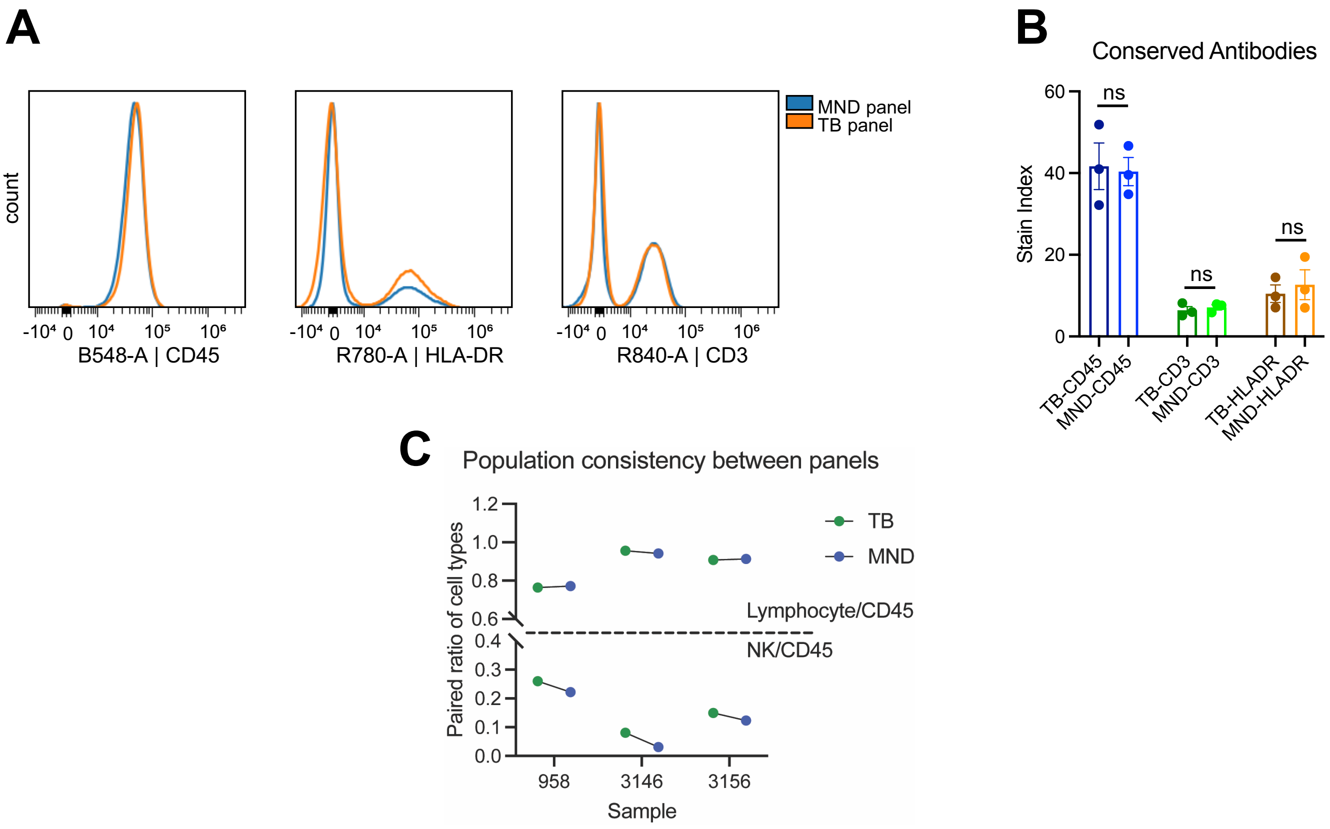

Figure S3. T/B and M/N/D Staining and Panel Consistency in Cell Type Detection. (**A**) Histograms showing expression of the 3 antibodies used in both the T/B and M/N/D PBMC panels (**B**) Bar graphs showing average stain index calculated using the median and standard deviation of the positive and negative peaks for each of the 3 antibodies used in both the T/B and M/N/D PBMC panels (n=3 donor samples). $Stain\ Index = \frac{(Median_{positive} - Median_{negative})}{2*SD_{negative}}$. Error bars represent SEM and statistical significance was calculated by t-test with p>0.05 being n.s. (not significant). (**C**) Ratio of lymphocytes (upper portion of graph, separated by the dotted line) and NK cells (lower portion of graph) to total CD45+ cells for each indicated donor in the T/B panel (green) and M/N/D panel (blue). Data were analyzed by two-way ANOVA with no significant differences calculated due to panel or cell type assessed.
