# Supplementary material for: Spectral Flow Cytometry Methods and Pipelines for Comprehensive Immunoprofiling of Human Peripheral Blood and Bone Marrow
Source: Cancer Res Commun. 2024 Mar 25;4(3):895–910. doi: 10.1158/2767-9764.CRC-23-0357 (PMC10962315; doi:10.1158/2767-9764.CRC-23-0357)
Supplement: Figure S4 — T Cell Activation and Exhaustion. Density plots of T cell activation and exhaustion markers on CD3+ PBMCs concatenated from three donor samples. [file crc-23-0357-s08.pdf]

Figure S4

CD3+ T Cell Activation and Exhaustion

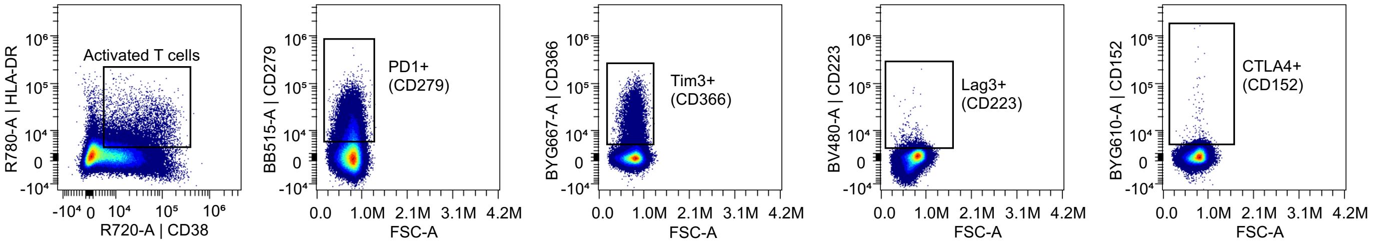

Figure S4. T Cell Activation and Exhaustion. Density plots of T cell activation and exhaustion markers on CD3+ PBMCs concatenated from three donor samples.
