# Supplementary material for: Spectral Flow Cytometry Methods and Pipelines for Comprehensive Immunoprofiling of Human Peripheral Blood and Bone Marrow
Source: Cancer Res Commun. 2024 Mar 25;4(3):895–910. doi: 10.1158/2767-9764.CRC-23-0357 (PMC10962315; doi:10.1158/2767-9764.CRC-23-0357)
Supplement: Figure S5 — NK cell Marker Expression. Density plots of NK cell functional marker expression by subtype. Columns depict NK cell subtype (all NK, Mature NK, Early NK, and Terminal NK cells), and rows show individual markers. The first row shows the gating strategy used to identify each NK cell subtype from the total NK cells parent population. Gates indicate positive populations, with the percentage of the parental population displayed in each panel. [file crc-23-0357-s09.pdf]

Figure S5

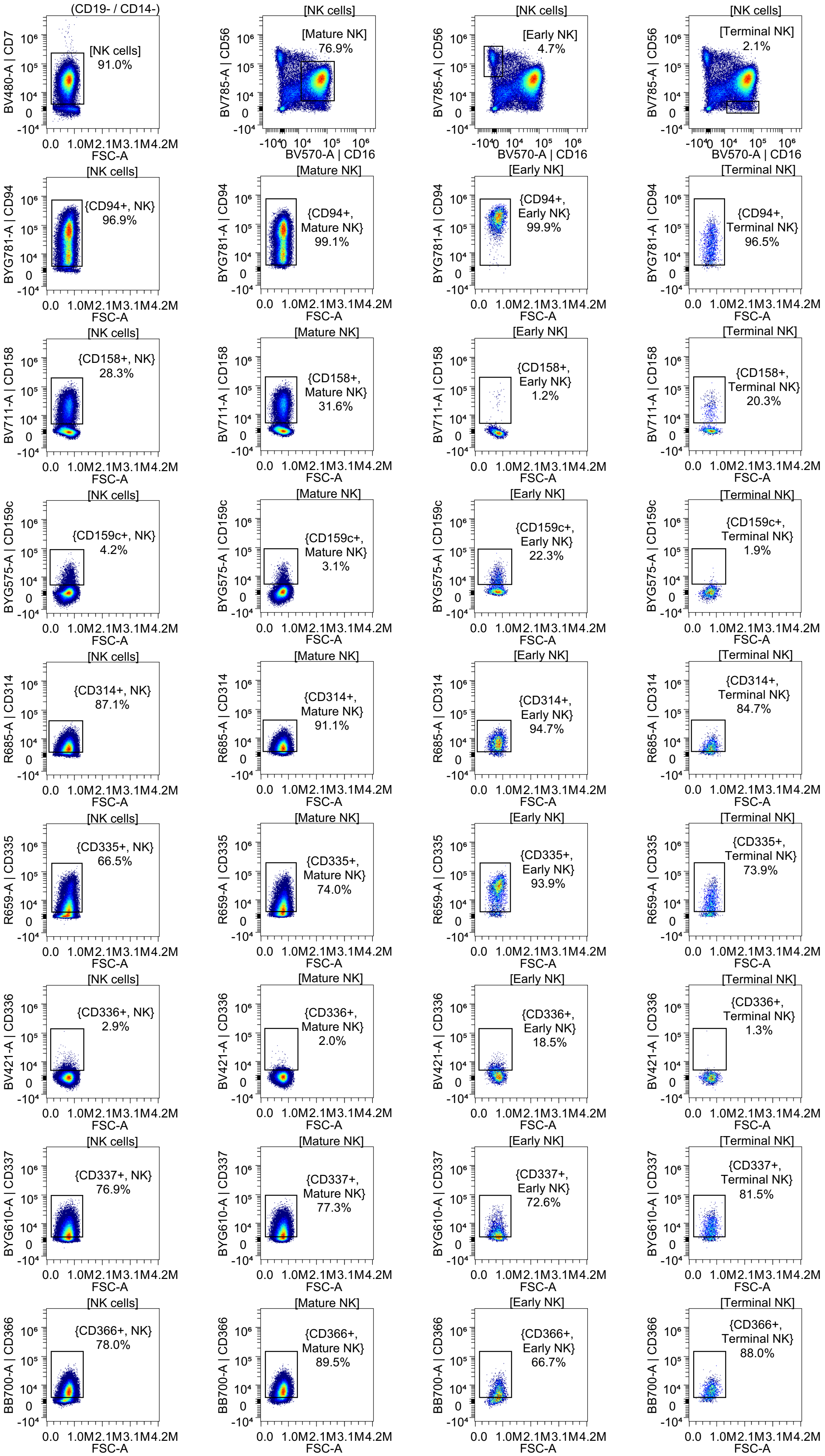

(CD19- / CD14-)
[NK cells]
91.0%
BV480-A | CD7
FSC-A
[NK cells]
[Mature NK]
76.9%
BV785-A | CD56
BV570-A | CD16
[NK cells]
[Early NK]
4.7%
BV785-A | CD56
BV570-A | CD16
[NK cells]
[Terminal NK]
2.1%
BV785-A | CD56
BV570-A | CD16
[NK cells]
{CD94+, NK}
96.9%
BYG781-A | CD94
FSC-A
[Mature NK]
{CD94+, Mature NK}
99.1%
[Early NK]
{CD94+, Early NK}
99.9%
[Terminal NK]
{CD94+, Terminal NK}
96.5%
[NK cells]
{CD158+, NK}
28.3%
BV711-A | CD158
[Mature NK]
{CD158+, Mature NK}
31.6%
[Early NK]
{CD158+, Early NK}
1.2%
[Terminal NK]
{CD158+, Terminal NK}
20.3%
[NK cells]
{CD159c+, NK}
4.2%
BYG575-A | CD159c
[Mature NK]
{CD159c+, Mature NK}
3.1%
[Early NK]
{CD159c+, Early NK}
22.3%
[Terminal NK]
{CD159c+, Terminal NK}
1.9%
[NK cells]
{CD314+, NK}
87.1%
R685-A | CD314
[Mature NK]
{CD314+, Mature NK}
91.1%
[Early NK]
{CD314+, Early NK}
94.7%
[Terminal NK]
{CD314+, Terminal NK}
84.7%
[NK cells]
{CD335+, NK}
66.5%
R659-A | CD335
[Mature NK]
{CD335+, Mature NK}
74.0%
[Early NK]
{CD335+, Early NK}
93.9%
[Terminal NK]
{CD335+, Terminal NK}
73.9%
[NK cells]
{CD336+, NK}
2.9%
BV421-A | CD336
[Mature NK]
{CD336+, Mature NK}
2.0%
[Early NK]
{CD336+, Early NK}
18.5%
[Terminal NK]
{CD336+, Terminal NK}
1.3%
[NK cells]
{CD337+, NK}
76.9%
BYG610-A | CD337
[Mature NK]
{CD337+, Mature NK}
77.3%
[Early NK]
{CD337+, Early NK}
72.6%
[Terminal NK]
{CD337+, Terminal NK}
81.5%
[NK cells]
{CD366+, NK}
78.0%
BB700-A | CD366
[Mature NK]
{CD366+, Mature NK}
89.5%
[Early NK]
{CD366+, Early NK}
66.7%
[Terminal NK]
{CD366+, Terminal NK}
88.0%
FSC-A

Figure S5. NK cell Marker Expression. Density plots of NK cell functional marker expression by subtype. Columns depict NK cell subtype (all NK, Mature NK, Early NK, and Terminal NK cells), and rows show individual markers. The first row shows the gating strategy used to identify each NK cell subtype from the total NK cells parent population. Gates indicate positive populations, with the percentage of the parental population displayed in each panel.
