# Supplementary material for: Spectral Flow Cytometry Methods and Pipelines for Comprehensive Immunoprofiling of Human Peripheral Blood and Bone Marrow
Source: Cancer Res Commun. 2024 Mar 25;4(3):895–910. doi: 10.1158/2767-9764.CRC-23-0357 (PMC10962315; doi:10.1158/2767-9764.CRC-23-0357)
Supplement: Figure S6 — Detection of Rare and Unexpected Cell Populations. Density plots of CD4+/CD8+ double-positive T cells (A), Basophils (B) and ILCs (C) identified in the PBMC samples. Gates indicate positive populations, with the percentage of the parental population displayed in each panel. Both concatenated and individual donor plots are shown. [file crc-23-0357-s10.pdf]

Figure S6

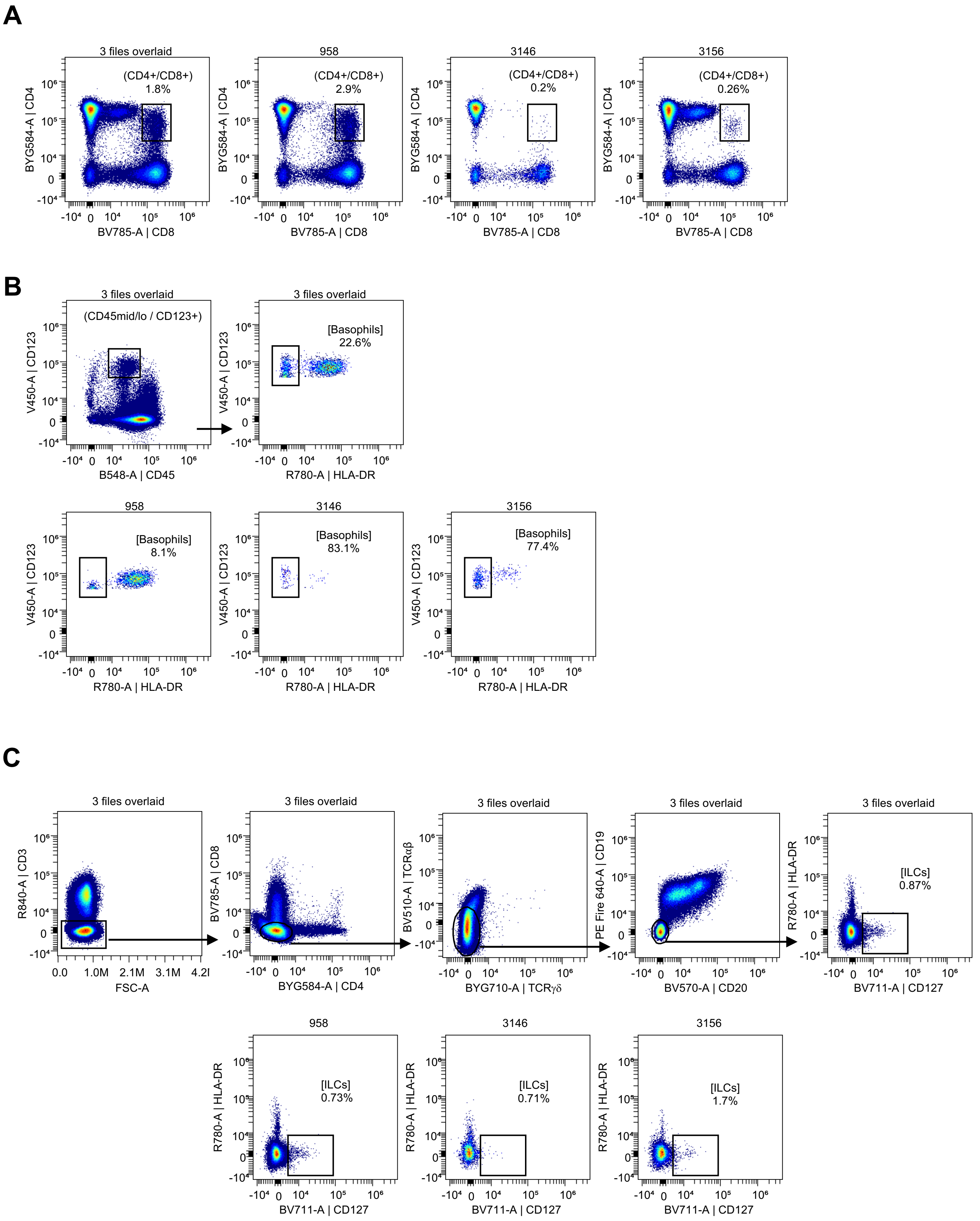
A
3 files overlaid
958
3146
3156
(CD4+/CD8+)
1.8%
2.9%
0.2%
0.26%
BYG584-A | CD4
BV785-A | CD8
B
(CD45mid/lo / CD123+)
[Basophils]
22.6%
8.1%
83.1%
77.4%
V450-A | CD123
B548-A | CD45
R780-A | HLA-DR
C
R840-A | CD3
FSC-A
BV785-A | CD8
BYG584-A | CD4
BV510-A | TCRαβ
BYG710-A | TCRγδ
PE Fire 640-A | CD19
BV570-A | CD20
[ILCs]
0.87%
0.73%
0.71%
1.7%
BV711-A | CD127

Figure S6. Detection of Rare and Unexpected Cell Populations.
Density plots of CD4+/CD8+ double-positive T cells (**A**), Basophils (**B**) and ILCs (**C**) identified in the PBMC samples. Gates indicate positive populations, with the percentage of the parental population displayed in each panel. Both concatenated and individual donor plots are shown.
