# Supplementary material for: Spectral Flow Cytometry Methods and Pipelines for Comprehensive Immunoprofiling of Human Peripheral Blood and Bone Marrow
Source: Cancer Res Commun. 2024 Mar 25;4(3):895–910. doi: 10.1158/2767-9764.CRC-23-0357 (PMC10962315; doi:10.1158/2767-9764.CRC-23-0357)
Supplement: Figure S7 — UMAP Marker Positioning. (A-E) UMAPs showing marker expression patterns in PBMC and BMC panels. Marker expression intensity is indicated by the scale bar to the right of each plot, where red is high, and blue is low. Data are derived from concatenated events from all 3 donors in the PBMC panels (A-D), and concatenated events from all 3 donors in the BMC panel (E). Insert in bottom right of each figure shows canonically gated major cell populations. Populations 10, 11, and 12 share CD16 expression, and as such occupy similar locations in UMAP space. [file crc-23-0357-s11.pdf]

Figure S7

A

T/B Panel – Lymphocytes

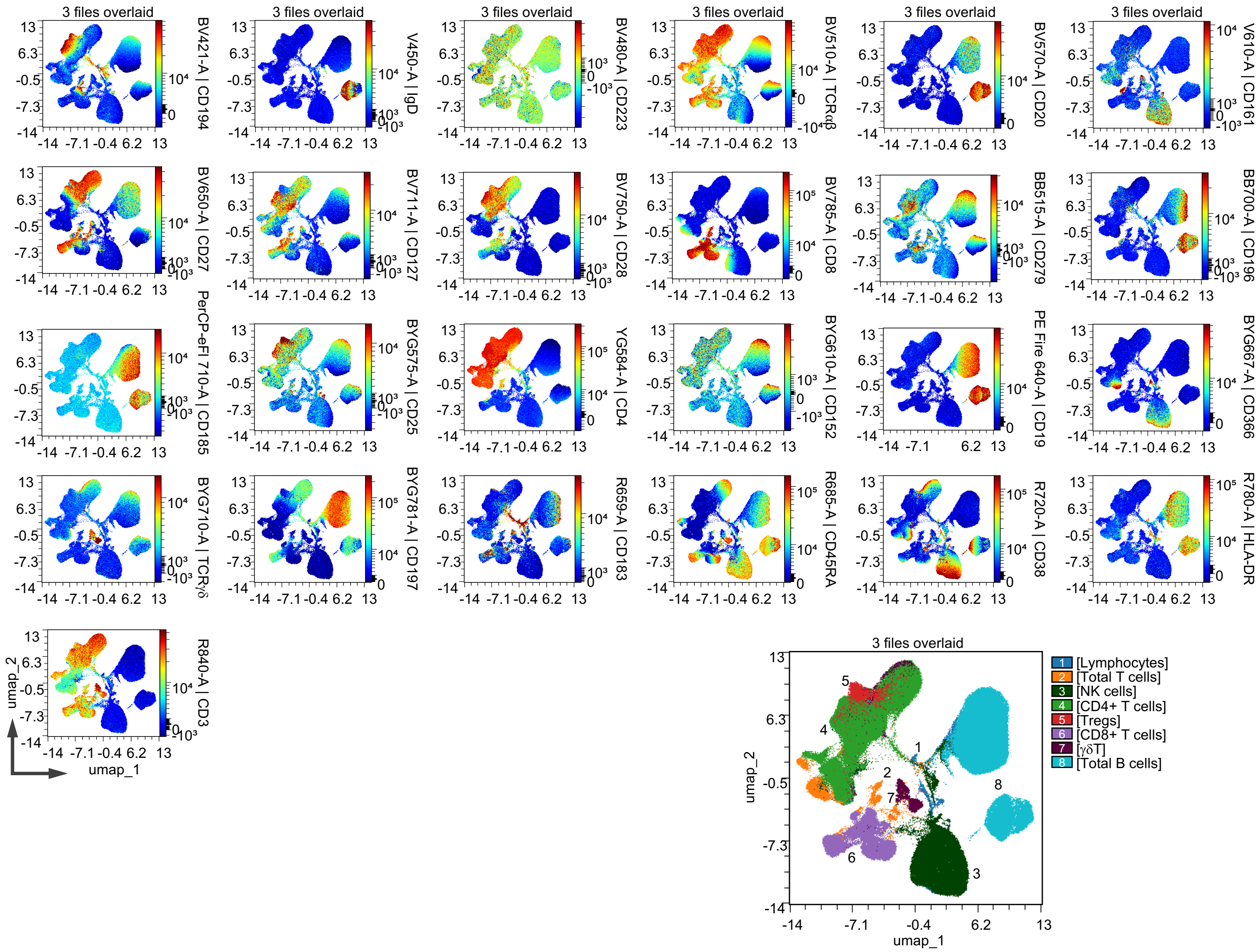

B

M/N/D Panel UMAP – All cells

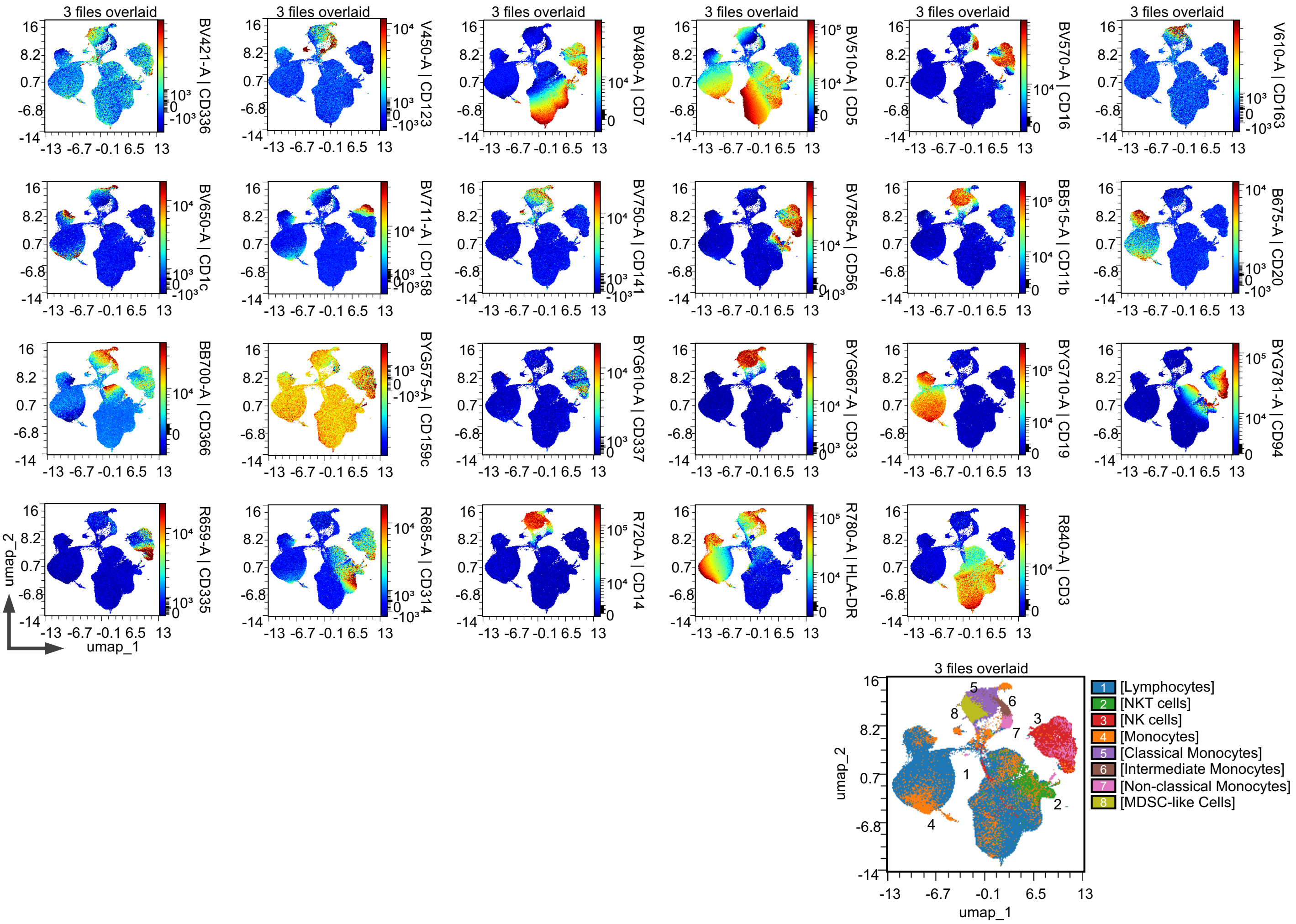

C

M/N/D Panel – Lymphocytes

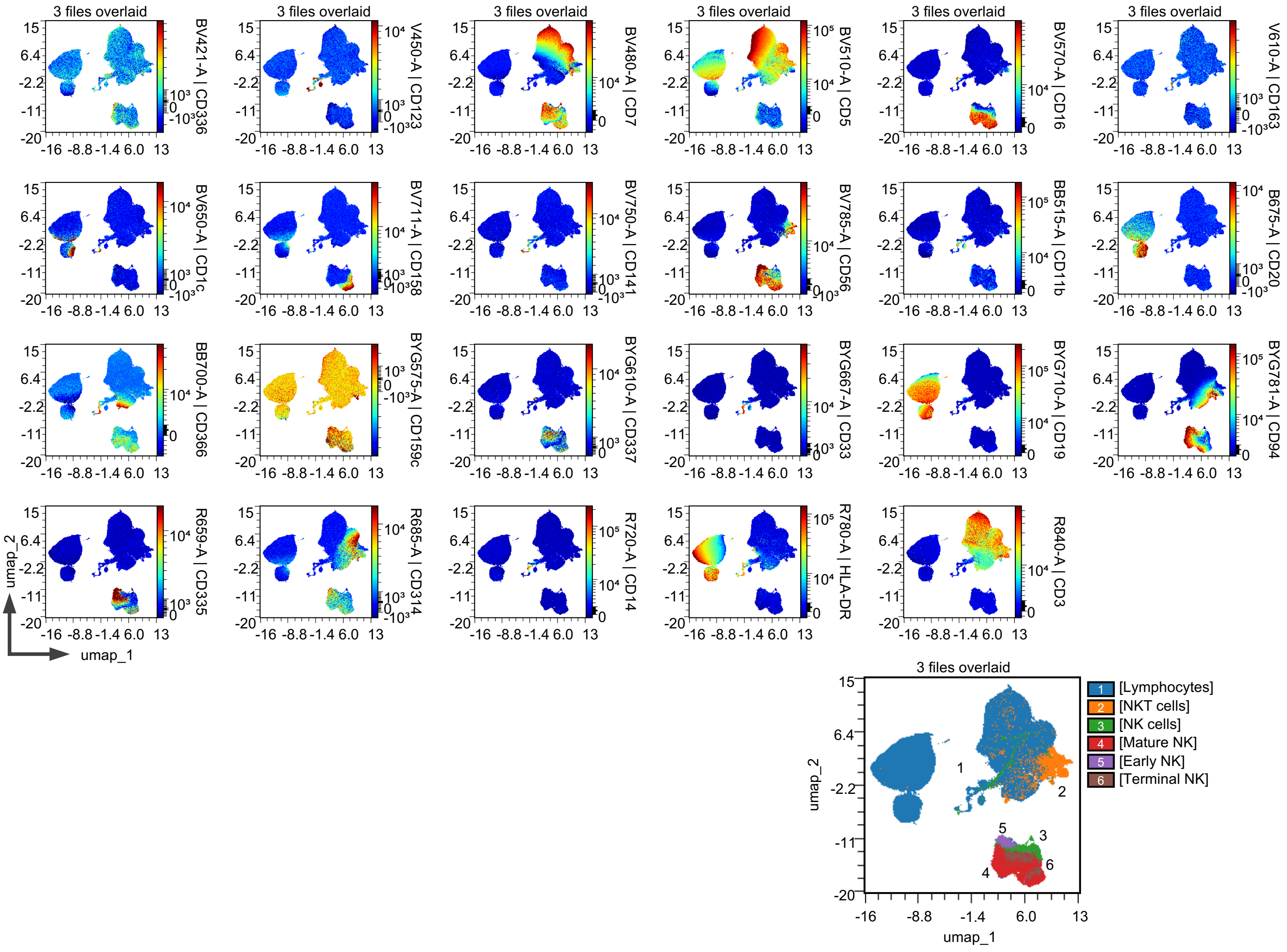

D

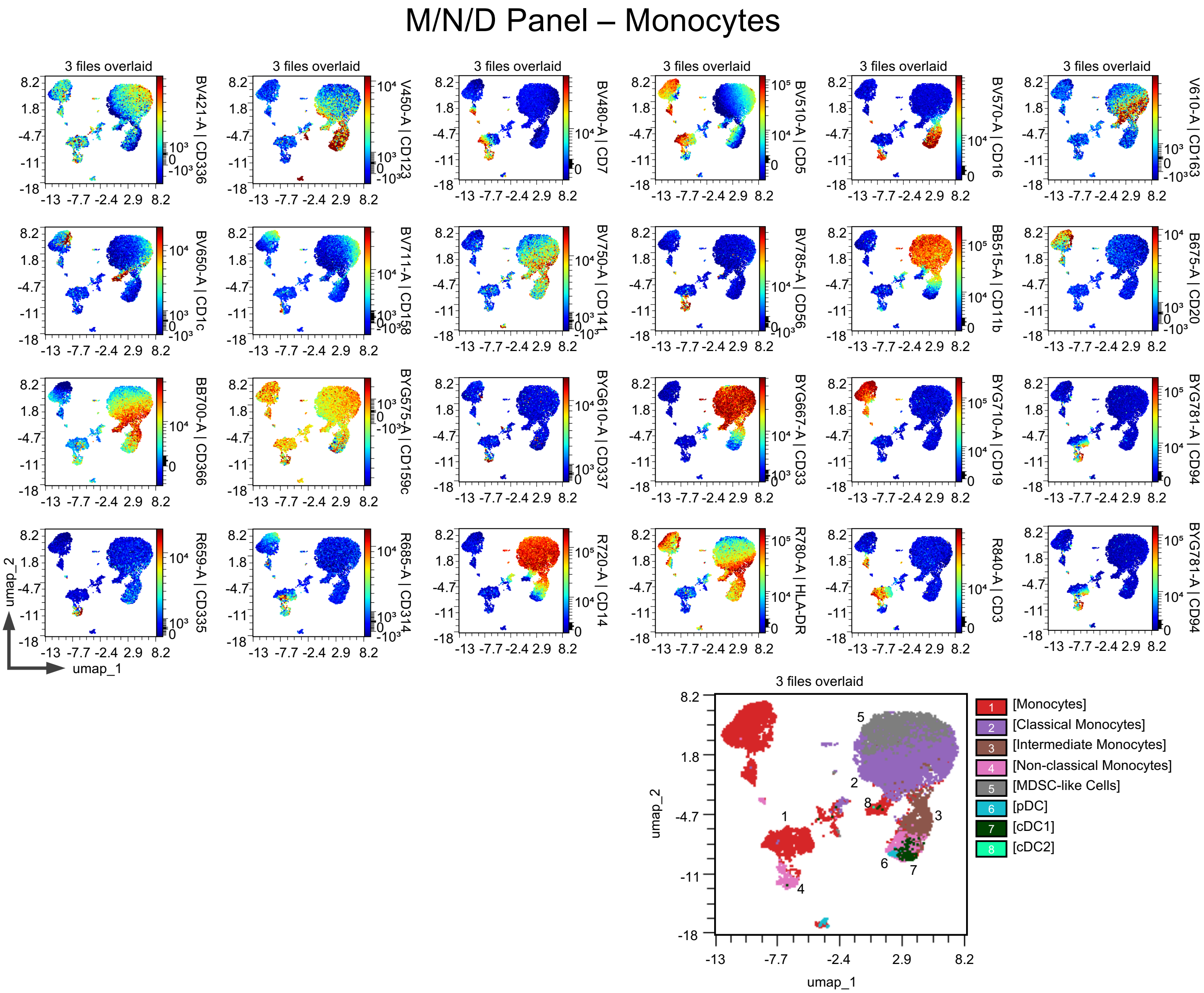
M/N/D Panel – Monocytes
3 files overlaid
BV421-A | CD336
V450-A | CD123
BV480-A | CD7
BV510-A | CD5
BV570-A | CD16
V610-A | CD163
BV650-A | CD1c
BV711-A | CD158
BV750-A | CD141
BV785-A | CD56
BB515-A | CD11b
B675-A | CD20
BB700-A | CD366
BYG575-A | CD159c
BYG610-A | CD337
BYG667-A | CD33
BYG710-A | CD19
BYG781-A | CD94
R659-A | CD335
R685-A | CD314
R720-A | CD14
R780-A | HLA-DR
R840-A | CD3
BYG781-A | CD94
umap_1
umap_2
[Monocytes]
[Classical Monocytes]
[Intermediate Monocytes]
[Non-classical Monocytes]
[MDSC-like Cells]
[pDC]
[cDC1]
[cDC2]

E

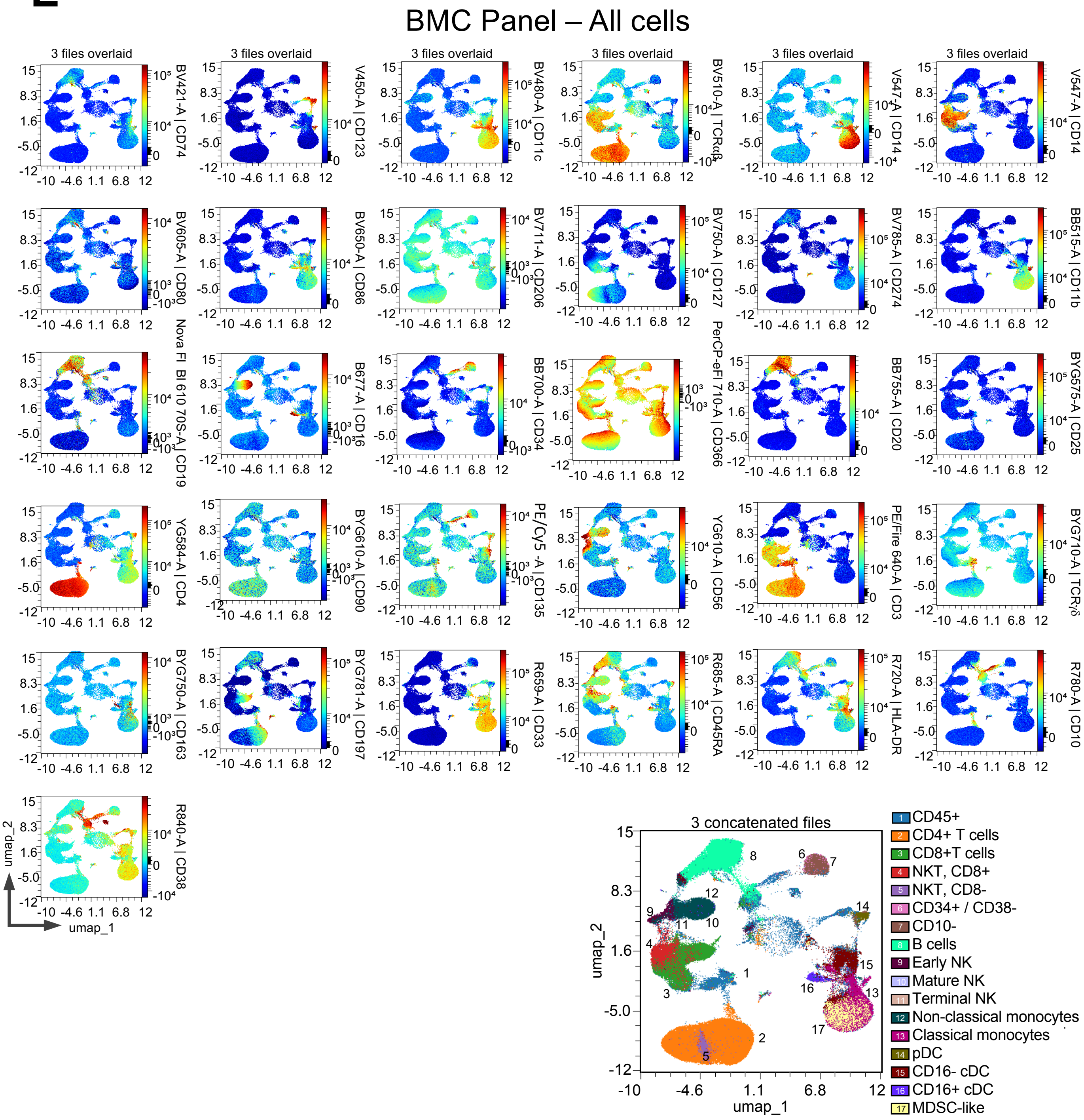

Figure S7. UMAP Marker Positioning. (**A-E**) UMAPs showing marker expression patterns in PBMC and BMC panels. Marker expression intensity is indicated by the scale bar to the right of each plot, where red is high, and blue is low. Data are derived from concatenated events from all 3 donors in the PBMC panels (**A-D**), and concatenated events from all 3 donors in the BMC panel (**E**). Insert in bottom right of each figure shows canonically gated major cell populations. Populations 10, 11, and 12 share CD16 expression, and as such occupy similar locations in UMAP space.
