# Supplementary material for: Spectral Flow Cytometry Methods and Pipelines for Comprehensive Immunoprofiling of Human Peripheral Blood and Bone Marrow
Source: Cancer Res Commun. 2024 Mar 25;4(3):895–910. doi: 10.1158/2767-9764.CRC-23-0357 (PMC10962315; doi:10.1158/2767-9764.CRC-23-0357)
Supplement: Figure S8 — Pre-integration LISI scores for PBMC and BMC UMAPs. Dot plot indicating proportion of cells with a high Local Inverse Simpson Index (LISI) score in concatenated UMAPs. To calculate this proportion, we set a threshold of LISI > 2 in those cases where scores could range from 1-3 (“BMC” and “PBMC (All Donors)”; UMAPs shown in Fig. 6A and 5A-D), or LISI > 1.667 in those cases where scores could range from 1-2 (“PBMC (Excluding Donor 3146)”, UMAPs not shown). Circular data points represent the proportion of LISI-high cells per indicated donor within each UMAP; red squares indicate the average proportion of LISI-high cells among all donor cells in the respective UMAP. For PBMC UMAPs, “All Donors” represents the inclusion of all three PBMC samples, while “Excluding Donor 3146” excludes a donor who had a B cell lymphoma, demonstrating how biological differences can impact integration. [file crc-23-0357-s12.pdf]

Figure S8

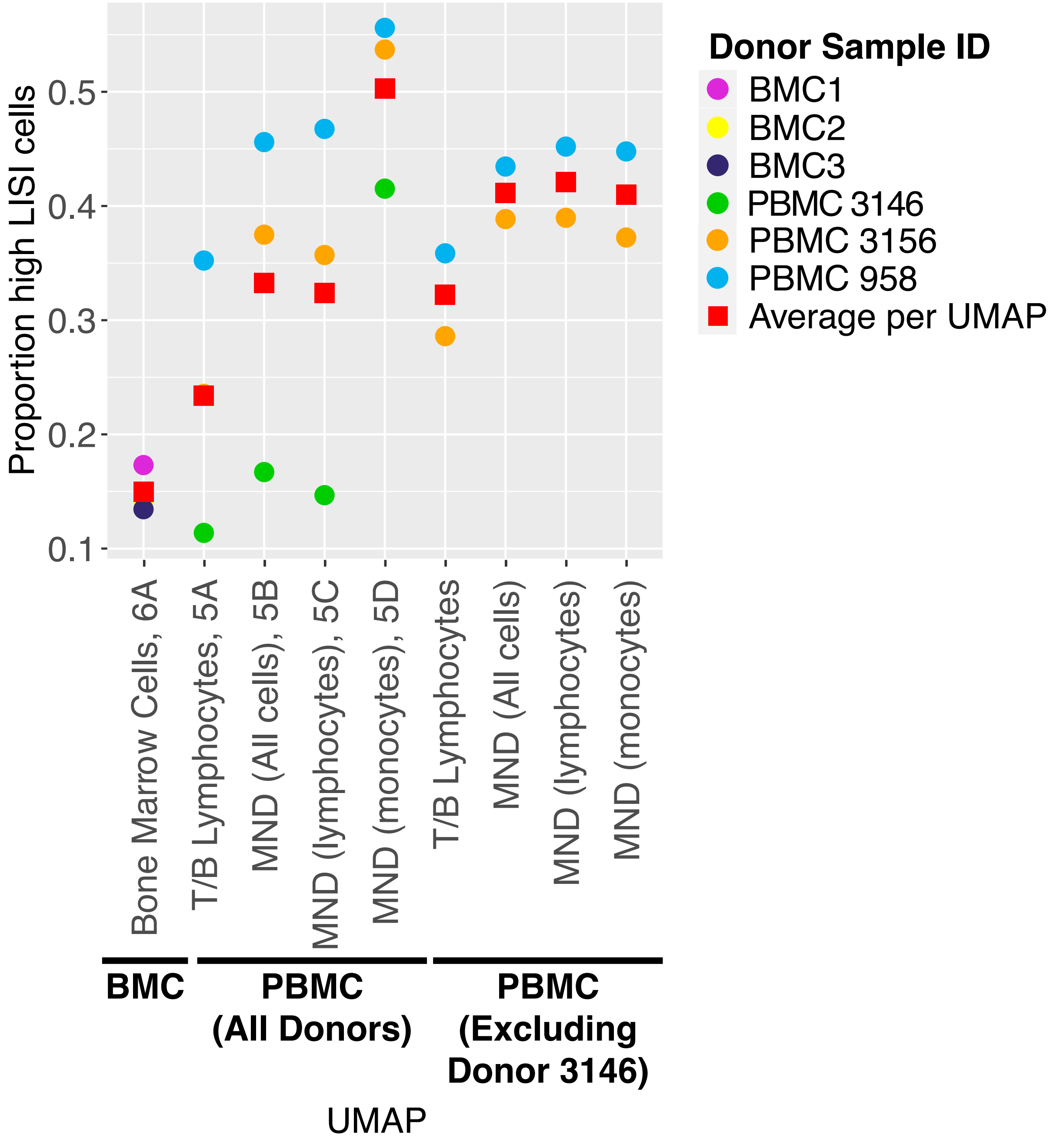

Figure S8. Pre-integration LISI scores for PBMC and BMC UMAPs. Dot plot indicating proportion of cells with a high Local Inverse Simpson Index (LISI) score in concatenated UMAPs. To calculate this proportion, we set a threshold of LISI > 2 in those cases where scores could range from 1-3 ("BMC" and "PBMC (All Donors)"; UMAPs shown in Fig. 6A and 5A-D), or LISI > 1.667 in those cases where scores could range from 1-2 ("PBMC (Excluding Donor 3146)", UMAPs not shown). Circular data points represent the proportion of LISI-high cells per indicated donor within each UMAP; red squares indicate the average proportion of LISI-high cells among all donor cells in the respective UMAP. For PBMC UMAPs, "All Donors" represents the inclusion of all three PBMC samples, while "Excluding Donor 3146" excludes a donor who had a B cell lymphoma, demonstrating how biological differences can impact integration.
